# Integrating Artificial Intelligence for Drug Discovery in the Context of Revolutionizing Drug Delivery

**DOI:** 10.3390/life14020233

**Published:** 2024-02-07

**Authors:** Anita Ioana Visan, Irina Negut

**Affiliations:** National Institute for Lasers, Plasma and Radiation Physics, 409 Atomistilor Street, 077125 Magurele, Ilfov, Romania; anita.visan@inflpr.ro

**Keywords:** artificial intelligence, drug discovery, machine learning, deep learning, drug repurposing, pharmaceutical AI

## Abstract

Drug development is expensive, time-consuming, and has a high failure rate. In recent years, artificial intelligence (AI) has emerged as a transformative tool in drug discovery, offering innovative solutions to complex challenges in the pharmaceutical industry. This manuscript covers the multifaceted role of AI in drug discovery, encompassing AI-assisted drug delivery design, the discovery of new drugs, and the development of novel AI techniques. We explore various AI methodologies, including machine learning and deep learning, and their applications in target identification, virtual screening, and drug design. This paper also discusses the historical development of AI in medicine, emphasizing its profound impact on healthcare. Furthermore, it addresses AI’s role in the repositioning of existing drugs and the identification of drug combinations, underscoring its potential in revolutionizing drug delivery systems. The manuscript provides a comprehensive overview of the AI programs and platforms currently used in drug discovery, illustrating the technological advancements and future directions of this field. This study not only presents the current state of AI in drug discovery but also anticipates its future trajectory, highlighting the challenges and opportunities that lie ahead.

## 1. Introduction

Drug discovery is a highly intricate and lengthy process that requires the identification of potential drug candidates that can effectively treat various diseases. The use of AI has brought a significant shift in the approach to drug discovery. AI has fundamentally transformed the pharmaceutical industry by speeding up the drug discovery process, improving precision, and decreasing costs. In this review, we will explore the different types of AI techniques used in drug discovery, including ML (to predict drug properties, identify potential drug candidates, and optimize chemical structures), DL (to analyze large-scale biological data, predict drug properties, and identify potential drug candidates), NLP (to analyze the scientific literature for potential drug candidates and to generate drug summaries), GM (to generate new molecules that could potentially be drug candidates), and network-based approaches (to identify potential targets for drug development).

### 1.1. Historical Background and the Concept of AI in Medicine

The use of artificial intelligence techniques, algorithms, and technologies in medicine and healthcare is referred to as AI in medicine. It involves the use of computer systems and specialized software to analyze medical data, make decisions, and perform tasks that are usually completed by human healthcare professionals. AI in medicine aims to improve the accuracy, efficiency, and effectiveness of medical diagnosis, treatment, and patient care by leveraging ML, NLP, and other AI methodologies. 

AI in medicine includes its diverse applications in areas such as medical image analysis, drug discovery, personalized treatment planning, disease diagnosis and prediction, virtual health assistants, electronic health record management, and patient monitoring. By processing vast amounts of patient data and medical literature, AI systems can assist healthcare professionals in making more up-to-date decisions, detecting patterns, and predicting patient outcomes, leading to better patient care and medical outcomes. The field of AI in medicine is rapidly advancing and making an impact in areas such as drug discovery, virtual health assistants, and remote patient monitoring. The use of AI-driven tools is expected to improve medical diagnoses, disease prevention, and treatment outcomes, ultimately leading to a more patient-centric and efficient healthcare system. The history of AI in medicine dates back several decades, with significant developments in both AI and medical sciences contributing to its evolution. Key milestones and events in the historical development of AI in medicine include the establishment of the field of AI in the 1950s by researchers like Alan Turing, who proposed the idea of intelligent machines [1]. Early work in AI involved attempts to mimic human problem-solving abilities through formal logic and rule-based systems [1]. In the 1960s, the concept of “expert systems” emerged, where the knowledge and expertise of human experts was encoded into computer programs to aid decision-making in specific domains [1]. This laid the foundation for AI applications in medicine. The development of early expert systems, such as Dendral and MYCIN, further advanced the use of AI in medicine. The 1970s saw an increase in AI applications in medicine, including computer-aided diagnosis (CAD) systems for medical imaging AI [1]. MYCIN, an expert system for diagnosing bacterial infections, demonstrated the potential of AI in healthcare [1]. In the 1980s, AI-based image recognition algorithms began to be applied in medical imaging, aiding in the interpretation of X-rays, CT scans, and MRIs [1]. AI techniques, such as pattern recognition and ML, were integrated into medical imaging systems [1]. The 1990s witnessed progress in NLP, enabling AI systems to “understand” and process medical text data [1]. Robotic surgery systems, such as the da Vinci Surgical System, were developed, combining AI and robotics for minimally invasive procedures [1]. With the advent of electronic health records (EHRs) and the growth of big data in healthcare, AI applications expanded to handle vast amounts of patient data [1]. AI in medicine began contributing to personalized medicine, predicting patient responses to treatments based on their individual characteristics [1]. DL, a subset of ML, revolutionized AI applications, including medical image analysis and disease diagnosis [1]. AI in medicine played a crucial role in advancing precision medicine, tailoring treatments based on genetic and molecular data [1]. These developments highlight the significant progress made in AI applications in medicine over the years, paving the way for improved healthcare outcomes and personalized treatment approaches. 

### 1.2. Statement of Significance

**Figure 1:**
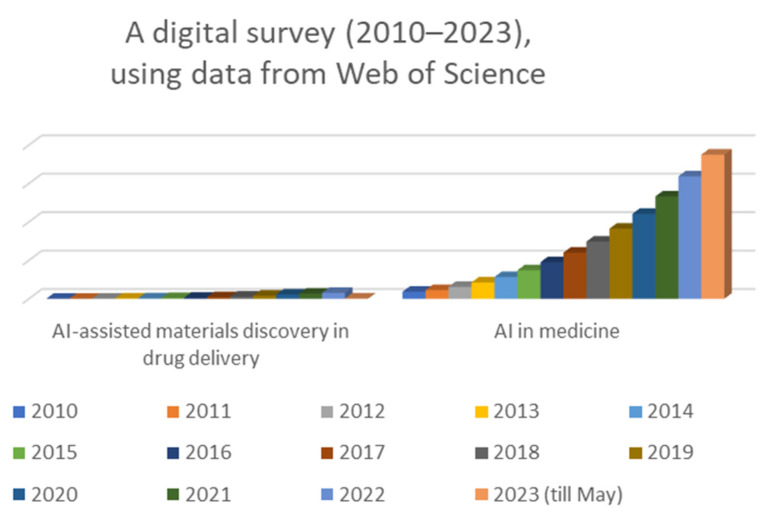
Digital survey (2010–2023) justifying the importance of the subject.

The historical background of AI in medicine shows a progressive journey from early explorations to the current era of sophisticated AI applications in various aspects of healthcare. As AI technologies continue to advance, they hold the potential to reshape the future of medicine and revolutionize healthcare practices for the benefit of patients and healthcare professionals alike. Overall, the number of published papers on AI has been increasing rapidly over time, with a peak of 348,684 papers being published in 2023. The number of published papers on AI-assisted material discovery in drug delivery has also been increasing in recent years, with a peak of 1234 papers being published in 2022. The number of published papers on AI in medicine has also been increasing in recent years, with a peak of 188,845 papers being published in 2021. The increase in the number of published papers on AI suggests that there is a growing interest in this technology and that more research is being conducted in this area. The number of published papers on AI in medicine has also been increasing in recent years but at a slower pace (Figure 1). The increase in the number of published papers on AI-assisted material discovery in drug delivery suggests that there is also a growing interest in the potential use of AI in this field. This suggests that there is still some way to go before AI is widely adopted into this field.

While the specific focus on AI-assisted material discovery in drug delivery is not extensively covered in the literature, the literature highlights the potential of AI in advancing pharmaceutical product development, including formulation design and drug discovery [2,3]. These insights suggest that AI can be a valuable tool in accelerating the discovery and optimization of materials for drug delivery. However, for a more detailed review specifically focusing on AI-assisted material discovery in drug delivery, it would be beneficial to explore additional research articles or publications that specifically address this topic.

## 2. AI in Discovering New Drugs

In the field of medicine, there are two types of AI applications: physical and virtual. Physical applications include the following: robot-assisted surgery, AI-enhanced prosthetics, real-time patient monitoring, and automated laboratory processes.

For example, AI in *robot-assisted surgery* can provide medical professionals with relevant information to assist them in making more informed decisions. While AI cannot replace human doctors, it can enhance their capabilities and improve patient care. Thus, AI-powered surgical robots enable surgeons to perform complex procedures with greater precision, control, and flexibility. These robots can reduce the risk of complications, minimize invasiveness, and shorten recovery times, leading to better surgical outcomes [4]. On the other hand, *AI-driven prosthetics* are designed to adapt to the user’s movements and respond to their neural signals. These advanced prosthetics significantly improve the quality of life for amputees, allowing them to perform complex tasks with greater ease and naturalness. *AI-based monitoring systems* continuously analyze patient data, such as their vital signs and electronic health records, to identify potential signs of deterioration or complications. This enables healthcare providers to intervene on time and avert adverse events. Studies have also shown that AI-based algorithms can outperform human doctors in certain diagnostic tasks, such as detecting certain types of cancer or interpreting pulmonary function tests [5]. Some *automated laboratory processes*, such as AI-powered robotic systems that streamline and automate laboratory processes, include sample analysis, sorting, and preparation. This reduces the workload for laboratory staff and minimizes the risk of human errors, ensuring more accurate results.

**Drug Discovery and Development** belong to the **Virtual Applications** category, together with diagnostic assistance, personalized treatment plans, and virtual health assistants. Virtual AI applications aid healthcare professionals in *diagnosing diseases* more accurately and efficiently. AI algorithms can analyze medical imaging data, such as X-rays, CT scans, and MRI images, to detect abnormalities and assist in early disease detection [6]. This capability translates to a significantly reduced chance of misdiagnosis and leads to better patient outcomes. The power of AI lies in its ability to process and analyze large amounts of medical data, spotting patterns that may not be immediately visible to humans. In doing so, AI can help improve diagnostic accuracy and develop personalized treatment plans. In particular, DL algorithms can identify anomalies or potential diseases in medical images, which can assist radiologists in their interpretations [6]. For instance, AI has proven useful in the field of gastroenterology by detecting abnormal structures in endoscopy and ultrasound images, such as colonic polyps [7]. Additionally, AI-powered wearable devices can remotely monitor patients and provide real-time data to healthcare professionals, offering early intervention opportunities. These devices have even been developed to detect and notify caregivers about seizures in epilepsy-suffering patients [8]. Also, AI-powered virtual applications can analyze an individual’s health data, including their genetics [9], medical history [10], lifestyle factors [11], and current health status, to create *personalized treatment plans*. These plans can optimize their medication dosage, predict their treatment response, and recommend targeted therapies, ensuring more effective and personalized patient care. AI can also contribute to precision medicine by analyzing a patient’s genetic data and medical history to predict their disease risk, determine optimal treatment plans, and identify potential drug targets. Moreover, *virtual health assistants*, powered by AI, offer patients 24/7 support and personalized health advice [12]. They can answer medical queries, remind patients about medication schedules, and provide lifestyle recommendations, promoting patient engagement and proactive healthcare management.

Regarding *drug discovery and development*, AI algorithms assist researchers in identifying potential drug candidates by analyzing vast databases of molecular structures, biological interactions, and clinical trial data. This accelerates the drug discovery process and holds promise for a faster development of new medications. 

AI in drug discovery implies:

 **(i)**  **Target Identification and Validation:**

**Figure 2:**
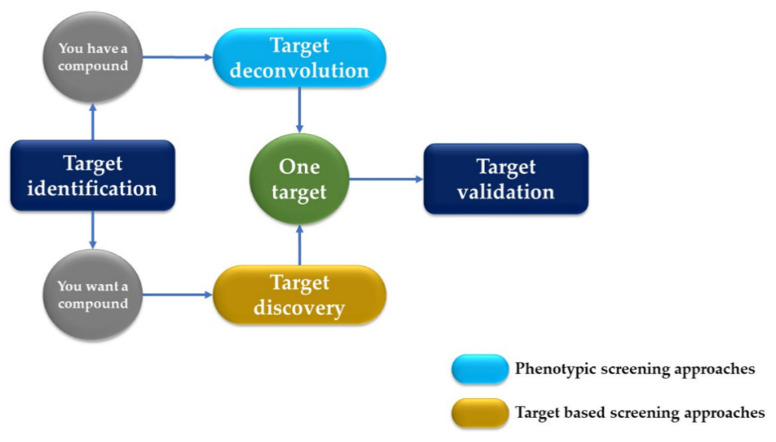
Drug development process: target deconvolution vs. target discovery.

AI has significantly impacted the field of drug discovery, particularly in the areas of target identification and validation. This process involves identifying potential biological targets and elucidating their roles in diseases, followed by validating these targets to ensure they are directly involved in a disease mechanism and that the modulation of the target is likely to have a therapeutic effect and plays a crucial role in identifying potential drug targets by analyzing the genomic, proteomic, and metabolomic data (Figure 2). ML algorithms sift through large datasets to pinpoint the proteins or biological pathways implicated in specific diseases, offering researchers valuable insights for drug development [13].

For instance, ML-based approaches, such as Kronecker regularized least squares (KronRLS), evaluate the similarities between drugs and protein molecules to determine DTBA. Correspondingly, SimBoost utilizes regression trees to predict DTBA, and considers both feature-based and similarity-based interactions [14].

AI also aids in the selection of the target. An optimal target should be druggable, safe, efficient, and able to fulfill commercial requirements. However, emerging modalities for disease treatment include previously less tractable targets. Target validation is a crucial step in drug discovery because it ensures that a molecular target is directly involved in a disease mechanism and that the modulation of the target is likely to have a therapeutic effect [15]. Target validation may involve determining the structure–activity relationship, the genetic manipulation of target genes (knockdown or overexpression), generating a drug-resistant mutant of the presumed target, using degradation-based tools to anticipate the effects of the target, and monitoring the signaling pathways downstream of the presumed target [16]. AI has been used to predict drug–target interactions [17], measure the binding affinity of a drug [18], and select and validate targets [19].

 **(ii)**  **Virtual Screening and Drug Design:**

AI-powered virtual screening tools analyze the three-dimensional structures of target proteins and predict how potential drug molecules would interact with them. This speeds up the process of drug designing and allows researchers to identify promising drug candidates for further testing [20].

**Figure 3:**
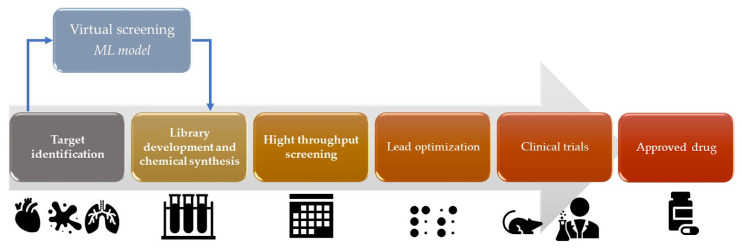
Virtual screening ML model in the drug development process.

Virtual drug screening is a computational approach that uses AI to predict the activity of potential drugs by fitting chemical structures to targets (Figure 3). This method allows researchers to rapidly test a library of compounds for their potential to bind and inhibit specific receptor or enzyme targets [21]. AI algorithms can analyze molecular structures, predict binding affinities, and prioritize compounds for further experimental testing [21]. Also, AI techniques, such as Bayesian docking approximations and RL, can be used in molecular docking simulations [22]. Molecular docking involves predicting the preferred orientation of a small molecule (drug candidate) when it is bound to a target protein. AI algorithms can explore the conformational space and predict the binding affinity between the drug and the target protein [22]. ML algorithms for drug design can be trained on/with large datasets of known drug–target interactions to envisage new drug–target pairs or optimize the properties of existing drugs. These algorithms can learn the patterns and relationships between chemical structures and biological activities, enabling the discovery of novel drug candidates [23]. For example, graph neural networks (GNNs) have been used to predict molecular properties and optimize drug design [24]. GNNs have been increasingly utilized in drug discovery for their ability to accurately predict molecular properties. These advanced AI models excel in understanding the complex structures of molecules, enabling a more efficient and targeted drug design. By representing molecules as graphs with atoms as nodes and chemical bonds as edges, GNNs effectively analyze and predict how these molecular structures will behave, leading to optimized drug design processes. AI can enhance high-throughput virtual screening, which involves screening large databases of compounds to identify potential drug candidates. ML algorithms can analyze chemical features, structure–activity relationships, and other molecular properties to prioritize compounds with high potential for further investigation [25]. 

AI techniques, such as DL and quantum chemistry, can be used for predictive modeling and optimization in drug design. DL models can analyze vast amounts of data, including chemical structures, biological activities, and pharmacokinetic properties, to predict the efficacy and safety of potential drugs [26]. Quantum chemistry combined with AI can accelerate the exploration of chemical space and guide the design of new drug candidates [25]. It is important to note that while AI has shown promising results in virtual screening and drug designing, experimental validation is still necessary for the identified drug candidates. AI is a powerful tool that can assist researchers in the early stages of drug discovery, but it should always be complemented with traditional experimental methods and rigorous testing [23]. Overall, AI has the potential to significantly accelerate the drug discovery process, improve the efficiency of virtual screening, and enable the design of novel drug candidates with optimized properties. As research in AI and ML continues to advance, we can expect further advancements in virtual screening and drug design methodologies.

 **(iii)**  **Prediction of Drug Properties**

AI algorithms can predict the physicochemical properties of drug candidates, such as the solubility, bioavailability, and toxicity [16]. This helps in optimizing drug development by focusing on compounds with a higher chance of success, thus reducing costs and time. ML algorithms can be trained to predict drug properties using a database of known compounds. These algorithms learn to recognize patterns and correlations between the chemical structure of a compound and its physicochemical properties [20]. Once trained, these models can predict the physicochemical properties of new, untested compounds, aiding in the early stages of drug development [20]. For instance, ML models have been used to predict solubility, a crucial property for drug candidates [27]. Similarly, ML algorithms have been used to predict bioavailability, another critical property for drug candidates [28]. Toxicity prediction is another area where AI has shown promise. ML models have been used to predict the toxicity of compounds based on their chemical structures [29]. These models can help in early-stage drug discovery by identifying compounds that are less likely to be toxic, thereby reducing the risk of harm to humans during the testing phase [14].

 **(iv)**  **Repositioning of Existing Drugs**

**Figure 4:**
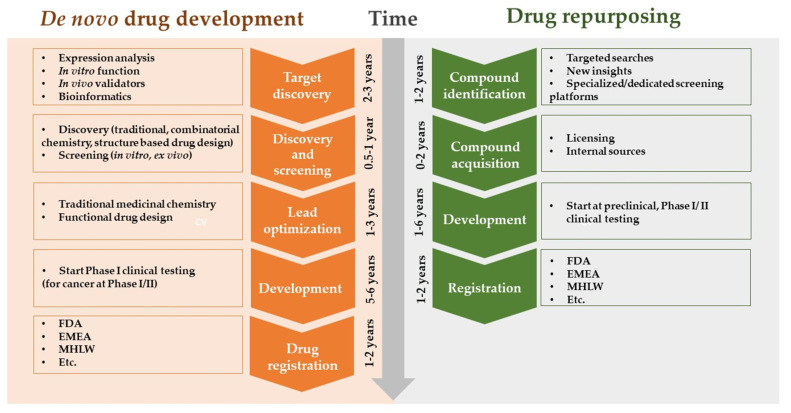
Drug repurposing approach.

AI enables researchers to identify new therapeutic applications for existing drugs by analyzing vast databases of drug–target interactions and disease pathways. This drug repurposing approach can significantly reduce the time and cost required to bring a drug to market (Figure 4) [30].

AI used in *drug combination identification* implies enhanced synergy detection, combination optimization, personalized combination therapies, and prediction of adverse drug interactions.

AI algorithms analyze high-dimensional biological data to identify potential synergistic drug combinations. By exploring interactions between drugs and their targets, AI can predict combinations that exhibit enhanced therapeutic effects while minimizing adverse reactions [31]. AI can optimize drug dosage and scheduling within a combination to maximize its efficacy and reduce side effects [32]. This fine-tuning ensures that the combination’s therapeutic benefits are fully realized, making it a more viable treatment option. AI-driven precision medicine allows the identification of patient-specific drug combinations based on individual molecular profiles and disease characteristics [33]. This personalized approach aims to achieve better treatment outcomes and minimize the risk of drug resistance. AI models can predict potential adverse interactions between drugs in a combination, ensuring the safety of patients and avoiding potential harm [34].

### 2.1. AI Techniques Used in Material Discovery

AI techniques, particularly in materials science, focus on discovering and designing new materials with desirable properties. This is directly applicable to drug discovery, as the process often requires the identification of novel compounds with specific biological activities. AI algorithms that excel in predicting the properties of new materials can similarly predict the pharmacological profiles of drug candidates. This crossover allows for the more efficient screening and optimization of potential drugs, leveraging AI’s predictive capabilities to streamline both the material and drug discovery processes.

AI techniques in materials discovery include supervised and unsupervised learning. Supervised learning uses labeled data to train models that can classify or predict outcomes of new data. Unsupervised learning, on the other hand, deals with unlabeled data and aims to develop models that can identify recurring patterns and clusters of the input data without prior knowledge. In contrast, in drug discovery, AI techniques are used at various stages, including in data collection and curation, compound representation, and AI methods and their applications. Data resources, data representation schemes, and AI methods are the three key components of applying AI to drug discovery and evaluation. AI techniques used in drug discovery include regression analysis, the decision tree, logistic regression, the support vector machine, the convolution neural network, the recurrent neural network, the generative adversarial network, k-means clustering, hierarchical clustering, principal component analysis, and t-distributed stochastic neighbor embedding.

In drug discovery, the selection and application of AI techniques are problem-oriented to ensure they are ideal. Two commonly used types of AI techniques in the realm of drug discovery are supervised and unsupervised learning [35].

Table 1 lists the widely used AI techniques in drug discovery, which are briefly discussed in the following sections. 

#### 2.1.1. Supervised Learning Methods

*Supervised learning* involves using input-labeled data to train models capable of classifying and/or predicting outcomes for new data, e.g., where the output is its corresponding property (e.g., band gap, thermal conductivity) [46]. Supervised learning models can then predict the properties of new materials based on *the patterns learned during training* [47]. In contrast, unsupervised learning deals with unlabeled data and aims to advance models which identify recurring patterns and clusters within the input data without prior knowledge [48]. They can help identify novel material classes that might not be apparent through manual analysis [49]. Supervised learning techniques are further split into classification and regression algorithms, while unsupervised learning techniques include clustering and dimensionality reduction algorithms. 

Linear regression models the relationship between a dependent variable and one or more independent variables by fitting a linear equation to the observed data. In drug discovery, linear regression can be used to predict a physicochemical property of a compound based on its molecular descriptors. Ridge regression and lasso regression are types of linear regression that use shrinkage, which is a technique for reducing the complexity of a model. SVR is a type of regression analysis that uses the concept of support vectors. It is a flexible method for regression and can handle both linear and non-linear problems. It can also handle high-dimensional data and is effective in high-noise situations. In material discovery, *reinforcement learning* (RL) can be applied to optimize the synthesis process [50]. Reinforcement Learning (RL) is an area of ML where an agent learns to make decisions by performing actions in an environment to achieve some goals. The agent receives feedback in the form of rewards or penalties, guiding it to learn the best strategy, known as a policy. RL involves trial and error, where the agent explores various actions and learns from the outcomes to optimize its behavior. This method is particularly useful in scenarios where explicit instructions on how to achieve a goal are not available, allowing the agent to autonomously discover effective strategies through iterative learning and adaptation. This approach is gaining traction in various fields, including robotics, gaming, and autonomous vehicles, due to its ability to adapt and improve through experience [50]. Two common RL algorithms are Q-learning and deep Q-Networks (DQNs). Q-learning is a model-free reinforcement learning algorithm that learns the value of an action in a particular state without requiring a model of the environment. A DQN combines deep learning with Q-learning to allow the agent to learn directly from raw pixels or other raw input. One of the key advantages of RL is that it focuses on the problem as a whole rather than dividing it into subproblems. It is capable of trading off short-term rewards for long-term benefits, and it does not require a separate data collection step, as training data are obtained via the direct interaction of the agent with the environment. To facilitate the application of these AI techniques, various open-source packages and frameworks have been developed, such as Scikit-learn [51], PyTorch [52], and Keras [53]. These packages provide access to a range of algorithms for users to practice using in drug discovery. Regression analysis algorithms play a crucial role in drug discovery, particularly in the prediction of the physicochemical properties of compounds. These algorithms can help optimize the drug development process by identifying compounds with desirable properties and reducing the time and cost of testing. 

*Regression analysis* is a statistical method used in drug discovery to predict the outcome of an experiment based on the values of predictor variables [54]. In the context of drug discovery, the outcome could be a physicochemical property of a compound, such as its solubility, bioavailability, or toxicity [54]. *Linear regression* is a basic and commonly used regression analysis method. It models the relationship between a dependent variable and one or more independent variables by fitting a linear equation to the observed data [55]. In drug discovery, linear regression can be used to predict a physicochemical property of a compound based on its molecular descriptors [56]. *Ridge regression* is a type of linear regression that uses shrinkage, which is a technique for reducing the complexity of a model [57]. The shrinkage parameter (or tuning constant) determines the amount of the shrinkage: the larger the value of the shrinkage parameter, the greater the amount of the shrinkage [58]. Lasso regression is another type of linear regression that uses shrinkage and can also handle high-dimensional data [59]. The main difference between ridge and lasso regression is that lasso can result in sparse solutions, where some of the feature coefficients are exactly zero, excluding those features from the model [60]. SVR is a type of regression analysis that uses the concept of support vectors [38]. It is a flexible method for regression and can handle both linear and non-linear problems. It can also handle high-dimensional data and is effective in high-noise situations [38].

*Classification* involves assigning data points to one of several possible categories. In drug discovery, classification algorithms can be used to predict whether a compound is likely to be effective or not.

A support vector machine (SVM) is a type of supervised learning algorithm used for classification and regression analysis. It works by finding a hyperplane in a high-dimensional space that distinctly classifies the data points. In drug discovery, SVM can be used to classify compounds based on their properties and predict their effectiveness.

A convolutional neural network (CNN) is a type of deep learning algorithm that is primarily used for image processing. However, it can also be used in drug discovery for tasks such as predicting the properties of new molecules. A CNN can automatically learn and extract features from raw data, making it suitable for handling complex and high-dimensional data.

A recurrent neural network (RNN) is a type of deep learning algorithm that is particularly suited for processing sequential data. In drug discovery, an RNN can be used to predict the properties of new molecules based on their sequence data.

A generative adversarial network (GAN) is a type of deep learning algorithm that consists of two neural networks, the generator and the discriminator, which are trained in an adversarial manner to generate realistic data samples. In drug discovery, GANs are applied to generate new molecular structures with desired properties.

#### 2.1.2. Unsupervised Learning Methods

*Unsupervised learning* involves using unlabeled data to identify patterns and relationships within the data. In drug discovery, unsupervised learning techniques can be used to identify novel material classes or to explore the chemical space of potential drug candidates.

Clustering (k-means) is a type of unsupervised learning technique that groups data points based on their similarity. In drug discovery, clustering can be used to identify groups of compounds with similar properties, which can help in identifying novel drug candidates.

Hierarchical clustering is a type of clustering technique that builds a hierarchy of clusters through either a bottom-up or top-down approach. In drug discovery, hierarchical clustering can be used to identify groups of compounds with similar properties at different levels of granularity.

Dimensionality reduction (PCA, t-SNE) techniques are used to reduce the number of variables in a dataset while retaining its essential characteristics. Principal component analysis (PCA) and t-distributed stochastic neighbor embedding (t-SNE) are two common dimensionality reduction techniques. In drug discovery, dimensionality reduction can be used to visualize high-dimensional data and identify patterns or trends that might not be apparent in the original data.

#### 2.1.3. AI Algorithms Used in Drug Discovery

While traditional approaches used in drug discovery rely heavily on manual research, experimentation, and testing, AI-driven methods/algorithms leverage data-driven analysis, ML, and rapid simulation.

##### Machine Learning and Deep Learning

ML and DL are subsets of AI that have found applications in drug discovery. While they share some similarities, they have distinct differences in terms of their approach, architecture, and capabilities [61].

(i) Machine Learning

ML is a broad field that encompasses various algorithms that can learn patterns and make predictions based on data. These algorithms typically work with structured data, and they require feature engineering, where relevant features are selected or engineered before feeding the data into the model [61,62,63]. Feature engineering is a critical step in preparing the data for ML models [63]. Traditional ML algorithms require well-structured and labeled data for training. ML algorithms are generally simpler and require less computational power compared to DL models [61,62]. They can perform well on certain tasks with limited data but may struggle with highly complex and non-linear problems. Traditional ML models may require a substantial amount of labeled data to achieve good performance, especially in complex tasks. Traditional ML models are generally more interpretable, meaning it is easier to understand how the model arrived at its predictions based on the selected features and parameters [61,62].

(ii) Deep Learning

On the other hand, DL is a specialized form of ML that uses artificial neural networks to learn representations of data [64]. These neural networks have multiple layers, allowing them to learn hierarchical features from raw data. Unlike traditional ML, DL models can automatically learn features from data, eliminating the need for extensive feature engineering [65]. DL models can handle unstructured data, such as images, texts, and sequences, without the need for extensive feature engineering. They learn hierarchies of representations directly from raw data, making them more suitable for handling complex and high-dimensional data [65]. DL models are more complex and require significant computational resources, especially when dealing with large datasets. They excel at handling complex patterns and non-linear relationships in data, making them particularly suitable for tasks like image and language processing [65]. DL models can often achieve better performance with less-labeled data due to their ability to automatically learn features and representations from the data. DL models are often considered less interpretable due to their multiple layers and complex learned representations. Understanding the decision-making process of DL models can be challenging. Gong D. et al. demonstrated how ML technology can be used to screen polymers for gene delivery in silico [66]. This work reported the application of state-of-the-art ML algorithms to a dataset of synthetic biodegradable polymers, PBAEs, which have shown promise for therapeutic gene delivery in vitro and in vivo [66]. The dataset included polymer properties as inputs as well as polymeric nanoparticle transfection performance and nanoparticle toxicity in a range of cells as outputs. These data were used to train and evaluate several state-of-the-art ML algorithms for their ability to predict transfection and understand structure–function relationships [66]. By developing an encoding scheme for vectorizing the structure of a PBAE polymer in a machine-readable format, the authors demonstrated that a random forest model can satisfactorily predict DNA transfection in vitro based on the chemical structure of the constituent PBAE polymer in a cell line-dependent manner [66]. Thus, a computational approach that encoded the chemical descriptors of polymers was able to demonstrate that the in silico computational screening of polymeric nanomedicine compositions had utility in predicting de novo biological experiments.

DL is a branch of AI that employs both supervised and unsupervised learning techniques, depending on the problem and data being analyzed [67].

(iii) High-throughput Density Functional Theory (DFT)

*High-throughput density functional theory* (DFT) calculations are computationally expensive but vital for understanding material properties at the atomic level [68]. High-throughput DFT involves using efficient algorithms and parallelization to predict the properties of a large number of materials rapidly [68]. DFT has become increasingly relevant in drug discovery, where it plays a crucial role in understanding and predicting the interactions between drug molecules and biological targets. 

High-throughput DFT can be used to calculate the binding energies and binding affinities between drug molecules and their target proteins [69]. These calculations help identify potential drug candidates with strong binding interactions, leading to a more efficient screening and selection of promising compounds. High-throughput DFT can be used in virtual screening to predict the binding affinities of a large library of drug-like compounds toward a specific target [70]. By virtually screening large numbers of compounds, researchers can prioritize the most promising candidates for further experimental validation. With the help of high-throughput DFT, researchers can investigate the nature of drug–target interactions at the molecular level [71]. It can identify key residues involved in binding and analyze the effects of ligand modifications on the binding affinity, which helps in the rational development of effective and selective drugs [72]. Algorithm high-throughput DFT can also predict physicochemical properties such as solubility, lipophilicity, and metabolic stability, which are crucial for assessing the pharmacokinetics and toxicity of a drug candidate [73]. These predictions guide the selection of compounds with favorable ADMET profiles and reduce the likelihood of failure in later stages of drug development. Also, high-throughput DFT can be used to study drug metabolisms by predicting the energy of various metabolic reactions such as hydroxylation or oxidation [74,75]. These predictions help identify potential metabolic sites and understand the metabolic pathways of drug candidates. On the other hand, high-throughput DFT can explore the conformational flexibility of ligands and identify the most stable conformers [76]. This information is critical for accurately representing ligand flexibility in molecular docking and molecular dynamics simulations and leads to more reliable binding predictions. High-throughput DFT can be used in fragment-based drug designing to analyze the binding of small fragments to a target protein. These calculations help select fragments that can be assembled into larger, more potent drug-like molecules [77].

(iv) Natural Language Processing (NLP)

*NLP* is a key AI technique used in text mining for drug discovery. NLP algorithms can process and interpret human language to extract relevant information from scientific literature, patents, clinical trial data, and other textual sources [78]. NLP-powered AI models can identify drug names, target proteins, chemical entities, and disease-related information, aiding researchers in gathering crucial data for drug discovery [78].

(v) Text mining

*Text mining* enables the identification of existing drugs with potential applications in different therapeutic areas [79]. By analyzing the literature, AI models can suggest drug candidates that have a demonstrated efficacy against specific diseases or targets, leading to drug repurposing opportunities and exploring the concept of polypharmacology [79]. AI models can integrate diverse data sources, such as clinical trial results, genomic data, and chemical databases, to build knowledge graphs [80]. Knowledge graphs represent complex relationships between drugs, targets, diseases, and biological pathways, facilitating comprehensive analysis and hypothesis generation [80]. Also, AI models can mine the literature to identify the adverse drug reactions reported in clinical studies and post-marketing surveillance. Extracting adverse drug reaction information from scientific literature helps improve drug safety assessments and informs decision-making in clinical trials [81]. Text mining by AI models can uncover the potential biomarkers associated with specific diseases or drug responses [82]. These biomarkers play a crucial role in personalized medicine, aiding in patient stratification and the development of targeted therapies. 

Various text mining-based tools have been harnessed through AI-driven techniques to leverage their capabilities. For instance, Jang et al. introduced PISTON (http://databio.gachon.ac.kr/tools/PISTON/ (accessed on 1 February 2024)), a tool that employs NLP and topic modeling to predict drug side effects and indications [83]. DisGeNET (https://www.disgenet.org/ (accessed on 1 February 2024)) is a text mining-driven database offering a wealth of information on gene–disease and variant–disease relationships [84]. DisGeNET’s data analysis encompasses diverse biological processes, including adverse drug reactions, molecular pathways involved in diseases, and drug actions on targets. Another tool is STRING (https://string-db.org/ (accessed on 1 February 2024)), which represents a text mining-driven database. It offers an abundance of information on protein–protein interactions across various organisms [85]. In addition, STITCH (http://stitch.embl.de/ (accessed on 1 February 2024)) provides valuable information on interactions between proteins and chemicals–small molecules [86]. STITCH’s information is also utilized to determine drug binding affinities and drug–target associations.

(vi) Generative Adversarial Networks (GANs)

*GANs* are a class of ML algorithms that consist of two neural networks, the generator and the discriminator, which are trained in an adversarial manner to generate realistic data samples [87]. In drug discovery, GANs are applied to generate new molecular structures [88] and optimize lead compounds [89], among other applications.

For example, GANs can generate novel molecular structures with certain desired properties. The generator network learns to produce realistic chemical structures, while the discriminator network evaluates their authenticity based on a training dataset. This process encourages the generator to produce new molecules that resemble the properties of known drugs, making GANs useful in de novo drug design [90].

Also, it was found that GANs can be employed to optimize lead compounds by generating modifications or analogs of existing drug candidates. By using the discriminator’s feedback to guide the generation of new chemical structures, GANs can propose modifications that are more likely to be biologically active and have improved pharmacological properties [91].

A number of studies have also shown that GANs can be used for multi-objective drug designing, where the goal is to optimize multiple drug properties simultaneously [41]. By adjusting the loss function of the GAN, researchers can balance various factors, such as the binding affinity, solubility, and selectivity, to generate molecules with desirable multi-objective profiles [41,89]. It should be noted that previous studies [92] have indicated that GANs can optimize specific molecular properties, such as the lipophilicity, molecular weight, or solubility. This capability allows researchers to fine-tune lead compounds or design molecules with the desired physicochemical properties [93].

According to the evidence to date, GANs can explore rare chemical space and identify molecules that are not commonly found in traditional drug databases. This capacity is valuable for discovering novel chemical entities with unique properties, potentially leading to the development of innovative drugs [94].

Studies show that GANs can be integrated into virtual screening pipelines to propose novel drug candidates. By generating diverse sets of molecules, GANs can expand the chemical diversity of virtual screening libraries and increase the chances of identifying hits against specific targets [95].

It should be noted that GANs can augment small datasets of known active compounds, enabling better generalization and improving the performance of predictive models by generating additional data samples [88]. Also, GANs enhance the efficiency and accuracy of the ML models used in drug discovery [96].

Present day science indicates that GANs can be used to predict drug–target interactions by generating molecular structures that are likely to interact with specific protein targets. The generated compounds can then be experimentally validated to identify potential novel drug–target interactions [94,97].

(vii) Transfer Learning

The other two AI algorithms used in drug discovery are *transfer learning* and *active learning*.

*Transfer Learning* makes it possible to fine-tune models that have been pre-trained on a larger dataset to cater to a specific material class or property using a smaller dataset. This is especially useful when there is a scarcity of labeled data. Transfer learning in drug discovery is an ML method that leverages knowledge gained from one task and applies it to another related task with limited data. This approach has shown great promise in addressing the challenge of sparsely labeled data in in silico drug discovery efforts [98]. In transfer learning, a pre-trained model developed for a specific task, such as image recognition, is used as a starting point for a new model targeting a different task, such as predicting drug–target interactions or identifying high-efficacy drug compounds [99]. By transferring the learned representations from the pre-trained model, the new model can benefit from the knowledge and generalization capabilities of the pre-trained model, even with a small amount of labeled data. In the past, quantitative structure–activity relationship (QSAR) models utilized regression models to establish links between molecule descriptors and biological properties [100]. 

In the field of drug discovery, ML techniques such as support vector machine algorithms and decision trees have been utilized for drug-like classification and the prediction of absorption, distribution, metabolism, excretion, and toxicity (ADME/T) properties [101,102,103]. Significant progress has been made in various areas through the use of AI technology, including predicting molecular properties and activities [104,105,106], virtual screening [107,108], retrosynthetic analysis [109,110], and generating new drugs [111,112]. Deep transfer learning, which involves using deep neural networks, is the most commonly used type of transfer learning in drug discovery [113]. Deep learning models, such as convolutional neural networks (CNN) and recurrent neural networks (RNN), have been applied to various drug discovery tasks, including compound activity prediction, virtual screening, and de novo drug designing [113]. These models can learn complex representations of chemical structures or biological data and transfer that knowledge to related drug discovery tasks. DL utilizes deep neural networks with multiple hidden layers to grasp and analyze complex knowledge, in contrast to traditional “shallow” ML approaches. Big data refers to datasets that are voluminous, diverse (i.e., originating from multiple sources), rapidly updated (i.e., in real-time), comprehensive (i.e., capturing the entirety of the system’s features rather than just samples), valuable (i.e., yielding numerous insights and potential for repurposing the data), and possess other defining characteristics [114]. Transfer learning offers a significant advantage in drug discovery by overcoming the challenge of limited and diverse datasets. Researchers can leverage pre-trained models to gain insights from vast and varied datasets in related fields [98]. For example, a pre-trained CNN model that has been trained on a large dataset of images can be fine-tuned and applied to analyze chemical structures or molecular fingerprints, enabling the prediction of compound activity or identifying potential drug–target interactions [98]. One useful application of transfer learning is its potential to speed up the process of discovering new drugs and enhance the effectiveness of identifying potent drug compounds. Through utilizing the knowledge stored in pre-existing models, researchers can decrease the amount of time and resources needed to train models from the ground up [98]. The method of using transfer learning in drug discovery enables faster testing and experimentation, which leads to speedy identification of promising drug candidates. Nevertheless, it is important to keep in mind that transfer learning in drug discovery is still an area of research with ongoing challenges and limitations to consider. One of these challenges is selecting the right pre-trained models and then fine-tuning them correctly. When choosing a pre-trained model, it is crucial to consider its relevance to the target task and the availability of appropriate datasets [35]. It is important to note that transfer learning does not always lead to better performance. This is particularly true when the source and target tasks are quite different. It is crucial to carefully assess how transferable the knowledge is between the tasks to ensure that the transferred representations are relevant and useful for the desired target task [98]. To sum up, transfer learning is a hopeful strategy in drug discovery which tackles the problem of insufficient labeled data and speeds up the detection of effective drug compounds. It involves using pre-trained models and applying knowledge from similar tasks to enhance the drug discovery process. This method can help researchers obtain useful representations and improve the efficiency of the process. However, there is still a need for more research to optimize the selection and fine-tuning of pre-trained models, and to explore the broader possibilities of transfer learning in drug discovery.

(viii) Active Learning

On the other hand, through the active selection of informative samples, *active learning* can determine which materials to test or simulate next, significantly reducing the number of experiments required. The use of AI techniques in material discovery has led to impressive advancements in drug discovery. By leveraging the power of computation and data-driven insights, researchers can more efficiently design and discover materials with customized properties for various applications. This ultimately accelerates innovation across industries [98]. Active learning is an ML technique used in drug discovery. Its goal is to improve the selection of compounds for experimental testing by iteratively selecting the most informative samples to label. This approach reduces the need for the exhaustive screening of large compound libraries and focuses on areas of chemical space with the highest potential for success while also considering structural novelty [115]. Active learning involves starting with a small group of labeled compounds and continuously choosing more compounds to label based on the model’s predictions. The selected compounds are then tested and the results are used to update the model and improve the selection process for future cycles [115]. By actively choosing which compounds to label, active learning algorithms can effectively navigate and utilize the chemical space, resulting in more efficient and cost-effective drug discovery processes. These algorithms strive for greater predictive accuracy using fewer labeled samples than traditional passive learning methods [115]. There are many drug discovery tasks that can benefit from active learning methods. These include predicting compound activity, optimizing leads, and virtual screening. Active learning can be used with various ML models, such as SVM, RF, and neural networks [115]. Active learning in drug discovery offers several benefits, including saving costs and resources. This is achieved by selecting a targeted subset of compounds for experimental testing, thereby reducing the need for extensive screening and saving time and resources [115]. Active learning algorithms can efficiently explore chemical space by choosing compounds that are likely to yield valuable information. This approach leads to a more targeted and effective drug discovery process [115]. Active learning can enhance the model’s predictive accuracy by regularly updating it with experimental feedback. This process leads to more precise predictions of compound activities over time [115]. When it comes to implementing active learning in drug discovery, there are some challenges and factors to consider. One of the biggest is choosing the right ML model and optimizing its parameters, which is crucial for success. It is important to find a model that can comprehend the complex connections between the chemical structures and activities, and the optimization process itself should be thoughtfully designed to balance exploration and exploitation. The selection of compounds for labeling is another vital step in active learning, and there are various strategies to consider, including uncertainty sampling, querying by committee, and diversity-based sampling. The best approach will depend on the task at hand and the data available. Finally, integrating active learning into existing drug discovery workflows may require some adjustments and considerations. The infrastructure, experimental design, automation, and data management should all be aligned with the active learning process to maximize its benefits [115]. To summarize, active learning is an effective method in drug discovery that leads to faster and more precise compound selection for testing. This is achieved by repeatedly selecting compounds based on the model’s predictions and experimental results. Active learning lowers expenses, enhances the exploration of chemical space, and improves predictive precision. However, it is crucial to thoughtfully select the models, data selection strategies, and workflow integration in order to successfully integrate active learning into drug discovery pipelines.

In the end of Section 2, we have centralized in Table 2 some collaborations between pharmaceutical companies and AI providers.

## 3. Studies of AI-Assisted Drug Discovery

**Figure 5:**
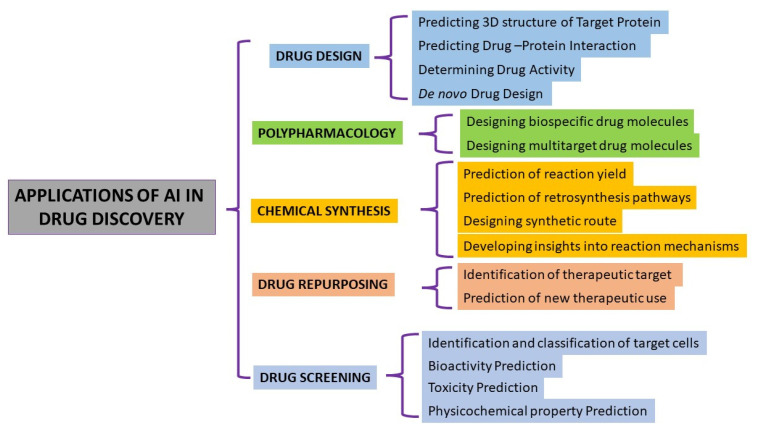
Applications of artificial intelligence in drug discovery.

AI offers a powerful tool for drug discovery, with applications ranging from predicting drug properties to generating new chemical structures and optimizing drug properties (Figure 5). One of the most common applications of AI in drug discovery is through the use of ML algorithms. These algorithms can learn from large datasets of chemical structures and biological data to predict the properties of new compounds, such as their ability to bind to a target protein (affinity prediction) or their toxicity (toxicity prediction). This can help to identify potential drug candidates that can be tested in the lab. Another application of AI in drug discovery is through the use of GANs that can be used to generate new chemical structures that are likely to bind to a target protein based on the chemical structures of known active and inactive compounds. AI can also be used in drug discovery for multi-objective optimization, where the goal is to optimize a drug for multiple desirable properties, such as high efficacy, low toxicity, and good solubility. 

### 3.1. AI Programs and Platforms Used for Drug Discovery

Different AI tools/platforms are already used in drug discovery (Table 3). 

For example, an MLP model uses a Python-based AI system to find a suitable candidate in drug discovery (DeepChem) and can be accessed at [116]. 

Another Python-based system driven by computational tools can aid the detection of the molecular activity of compounds (DeepNeuralNetQSAR) and can be found at [117].

On the other hand, there exists a program that helps to report the procedure for chemical synthesis in a standardized format (Chemputer) [118].

The Tox21 Data Challenge tested 12,000 chemicals for toxic effects using specially designed assays. DL was used in this example to predict toxicity. DeepTox software outperformed other methods, proving DL to be a superior method for toxicity prediction [119].

AlphaFold is an AI program developed by DeepMind, a subsidiary of Alphabet, that predicts the 3D structures of proteins. The program utilizes deep learning techniques and has been highly successful in protein structure prediction. AlphaFold participated in the Critical Assessment of Structure Prediction (CASP) competition and achieved remarkable accuracy in predicting protein structures, outperforming other groups [120]. AlphaFold has undergone two major versions: AlphaFold 1 (2018) and AlphaFold 2 (2020). In both versions, the program demonstrated exceptional performance in predicting protein structures. AlphaFold 2, in particular, achieved a level of accuracy much higher than any other group in the CASP competition. It scored above 90 for approximately two-thirds of the proteins in the global distance test (GDT), a measure of how closely the predicted structure aligns with the experimentally determined structure.

The success of AlphaFold has been regarded as a significant breakthrough in the field of structural bioinformatics. However, it is important to note that while AlphaFold has achieved remarkable accuracy, there are still challenges to be addressed, and the protein folding problem is not considered completely solved. Nonetheless, the technical achievement of AlphaFold has garnered widespread respect and has the potential to accelerate the advancement of structural bioinformatics. 

AlphaFold utilizes an AI-based approach called Evoformer, which treats protein structure prediction as a graph inference problem in 3D space. This approach involves updated operations applied in series within each block of the network. The pair representation in AlphaFold encodes information about the relationships between the residues, while the MSA (multiple sequence alignment) representation encodes individual residues and the sequences in which they appear. The MSA representation updates the pair representation through an element-wise outer product, allowing continuous communication between the evolving MSA representation and the pair representation. This approach enables the accurate prediction of protein structures [121]. The development of AlphaFold has the potential to revolutionize structural bioinformatics and accelerate the advancement of our understanding of protein structures. By combining AI techniques with curated structure and sequence databases, it can help bridge the gap between genomics and experimental structure determination, leading to a deeper understanding of biological processes and the development of new therapies [121].

ORGANIC [122] is a molecular generation tool that helps create molecules with certain desired properties. It utilizes generative ML approaches and deep neural networks to enable the design of novel molecular materials. The goal-directed generative model powered by deep neural networks and high-throughput simulations allow for a more directed design approach, minimizing design bias and enabling the exploration of specific material’s property spaces [123]. One of the key applications of ORGANIC is in the design of organic electronic materials, such as those used in organic light-emitting diodes (OLEDs). By leveraging generative ML frameworks, such as recurrent neural networks (RNNs) and deep RL, ORGANIC can rapidly identify new materials for OLED applications. This approach accelerates the development of novel organic electronic materials while also enabling expansion into other domains like catalyst design, aerospace, life science, and petrochemicals [123].

Another area where ORGANIC can be applied is in the virtual design of organic semiconductors based on metal–organic frameworks (MOFs). MOFs offer a supramolecular approach to modulate the arrangement of organic semiconductor molecules, allowing for the tailoring of the material properties. ORGANIC can automate the design process by generating MOF structures, sampling their structural dynamics, and predicting properties such as the conduction properties, absorption, and interaction with light. This automated workflow tool enhances the modeling and characterization of a wide variety of MOFs [124].

The development of ORGANIC provides researchers with a powerful tool for molecular generation and property optimization. By leveraging ML techniques and high-throughput simulations, it enables the rapid exploration of chemical space and the design of molecules with the desired properties. The availability of user-friendly interfaces and its integration with existing computational chemistry software, such as VASP and Gaussian, further enhances its usability and applicability in various research domains [125].

PotentialNet [126] is a program that utilizes neural networks to predict the binding affinity of ligands in protein–ligand complexes. It employs deep attention mechanisms and descriptor embeddings to improve the accuracy of binding affinity prediction [127]. The program focuses on intermolecular interactions within the molecular complex to capture important descriptors for an accurate prediction [127]. The approach used in PotentialNet involves representing the molecular complex as a 1D vector based on the descriptor information obtained from the training dataset. The descriptors are generated for the contacted protein and ligand atom pairs in the complex. The distance between the protein atom and the ligand atom pair is calculated using the Euclidean distance, with a cutoff value typically set at 12 Å [127]. The program considers various intermolecular interactions, including steric interactions, hydrophobic interactions, and hydrogen bonding.

The model architecture of PotentialNet incorporates an attention mechanism, which highlights important descriptors for a binding affinity prediction [128]. This mechanism helps capture the binding sites in the protein–ligand complex and improves the overall prediction performance. The program also utilizes a deep neural network model, specifically a PUResNet architecture, which consists of encoder and decoder blocks with skip connections [128].

To evaluate the performance of the binding affinity models, several metrics are used, including the mean absolute error (MAE), root mean square error (RMSE), Pearson’s correlation coefficient (PCC), Spearman’s correlation coefficient (SCC), and the standard deviation in regression (SD) [127]. The selection of an optimal number of descriptors is conducted by training a random forest model and sorting the descriptors according to their priorities. The model is then trained using different numbers of descriptors, and the performance is evaluated to determine the optimal number [127].

Hit Dexter is an ML technique used for predicting molecules that might respond to biochemical assays. It is an ML model specifically designed for hit discovery in the drug discovery process. Hit discovery involves identifying small molecules that have the potential to bind to a target and alter its function. By applying diverse algorithms and multivariate parameters, Hit Dexter aims to identify hits against specific biological targets or neurological complications [35].

One of the key components of Hit Dexter is the utilization of deep learning techniques to extract and analyze the chemical and physical properties of molecules. For example, deep learning models have been used to predict properties such as the absorption, distribution, metabolism, and excretion (ADME) of drug candidates. These models incorporate molecular graphs and utilize concepts like CNNs and ANNs to predict properties such as oral drug absorption. By considering various molecular descriptors and using ML algorithms like SVM regression and boosting, accurate predictions can be made [35].

Another aspect of Hit Dexter is the use of QSAR models. QSAR models leverage deep learning techniques to extract features from chemical strings and automatically extract relevant molecular descriptors. These models have shown promising results in hit-to-lead optimization research, providing predictions for binding affinities and aiding in the selection of potential drug candidates [35].

The application of Hit Dexter extends beyond drug discovery. It can also be used in the prediction of bioactivity, prognosis of prote–protein interactions, homology modeling/prediction of protein folding, and digital pathology [35].

DeltaVina [129] is a scoring function used for rescoring the binding affinity between proteins and ligands. It incorporates ML techniques and diverse algorithms to improve the accuracy of binding affinity predictions [130]. DeltaVina is designed to improve the accuracy of scoring the protein–ligand binding affinity. It utilizes various ML algorithms, including XGBoost and random forest, to incorporate different molecular descriptors and features. The performance of DeltaVina has been evaluated on benchmark datasets and compared to classical scoring functions, demonstrating its effectiveness in predicting binding affinities and identifying potential drug candidates. The scoring function and its code are available for use and further research [131]. 

The neural graph fingerprint [132] is a technique used to predict the properties of novel molecules. It involves generating molecular sentences and encoding them using a language model, such as BERT, to obtain high-dimensional embeddings of the substructures. These embeddings represent the molecules in vector form, which can be used for downstream tasks like molecular property prediction [133].

Once the molecular sentences are constructed, they are encoded using a language model, such as BERT. This pre-trained model can generate high-dimensional embeddings of the substructures, representing the molecules in vector form. These embeddings capture important features and structural information of the molecules, which can be used for predicting their various molecular properties [133]. To evaluate the performance of the prediction model, different metrics are used. For example, for classification tasks, the cross-entropy loss function is used, and the prediction performance is evaluated using the ROC-AUC (the area under the receiver operating characteristic curve) [133]. The ROC-AUC represents the ability of the model to distinguish between positive and negative samples. On the other hand, for regression tasks, the mean squared error (MSE) loss function is used, and the prediction performance is evaluated using the root mean squared error (RMSE) and the coefficient of determination (R^2^) [133]. The RMSE measures the average squared difference between the predicted and real property values, while the R^2^ reflects the goodness of fit and prediction. The neural graph fingerprint, with its molecular sentence generation and language model-based encoding, offers a powerful approach for predicting the properties of novel molecules. By leveraging deep learning techniques and molecular embeddings, it enables accurate predictions and the analysis of various molecular properties [133].

GastroPlus [134] is a platform that utilizes AI and predictive modeling to aid in the design and optimization of drug formulations. This software is used for pharmaceutical products (dosage form) in many animal models, including rats, primates, and animals, allowing researchers to predict the drug absorption, distribution, metabolism, and excretion [135]. GastroPlus’s AI-driven approach aids in the development of more effective and bioavailable drug formulations, streamlining the drug development process.

### 3.2. Example of AI in Drug Design

Next, we will present some examples which cover a range of topics related to AI in drug design, including the integration of AI in drug discovery, the prediction of drug–protein interactions, and practical guidelines for using ML algorithms. They provide valuable insights into the use of AI in the pharmaceutical industry and offer potential solutions to challenges in drug design and development.

For example, Paul D. et al. discusses the integration of artificial intelligence in drug discovery and development, including the tools and techniques used. They also highlight ongoing challenges and ways to overcome them [14].

Dara S. et al. explores various applications of artificial intelligence in drug design, including in the prediction of drug–protein interactions, the discovery of drug efficacy, and ensuring safety biomarkers [35].

Jiménez-Luna et al. focuses on explaining compound activity predictions using a substructure-aware loss for graphing neural networks. They explore the use of deep learning in predicting molecular properties [24]. They also provide practical guidelines for using gradient boosting in molecular property prediction. They offer insights into the application of ML algorithms in drug design.

Schneider et al. discuss the foundation of artificial intelligence in therapeutic science, including its applications in drug design. They explore adaptive graph learning methods for automated molecular interactions and property predictions [136].

The iterative process to design the 3D structures of receptors to generate a novel molecule is termed de novo drug designing, which is intended to produce new dynamics. Researchers utilized the indolent space portrayal to prepare a model dependent on the quantitative estimate of the drug likeness and drug similarity scores, and the manufactured availability score and synthetic accessibility score [137]. 

### 3.3. Examples Cases of AI Used in Polypharmacology

Next, we present some examples of papers that shed light on the application of AI in polypharmacology and drug discovery. They discuss how AI can be used to predict drug–protein interactions and design safer drug molecules. Understanding polypharmacology is crucial in developing drugs that are effective against multiple targets while minimizing off-target effects. These papers provide valuable insights into the advancements and challenges in this field and offer potential strategies for improving drug design and development. Chauhari et al. explain the concept of polypharmacology, which refers to the tendency of a drug molecule to interact with multiple receptors, leading to off-target effects. They explore how artificial intelligence can aid in the design of safer drug molecules by considering polypharmacology [138]. 

Reddy et al. [139] provide an overview of polypharmacology studies and the challenges involved. They discuss the potential of a rational design using polypharmacology to develop more effective and less toxic therapeutic agents. The review outlines the latest progress in this field. Other authors [140] explore polypharmacy using AI and data analysis. The AI axis focuses on defining and detecting polypharmacy using algorithms to predict health outcomes based on medication combinations.

The use of AI in pharmaceutical product development is also discussed in a review [14]. The review suggests that AI can replace the traditional trial-and-error method in designing formulations. Computational tools, including quantitative structure–relationship (QSPR) models and decision-support tools, can help address issues related to formulation design such as stability, dissolution, and porosity. Furthermore, the integration of AI with mathematical models like computational fluid dynamics (CFD) can speed up the production of pharmaceutical products [14].

The involvement of AI in the development of a pharmaceutical product from the bench to the bedside can be imagined given that it can aid rational drug design [141]; assist in decision making; determine the right therapy for a patient, including personalized medicines; and manage the clinical data generated and use it for future drug development [142]. E-VAI is an analytical and decision-making AI platform, developed by Eularis, which uses ML algorithms along with an easy-to-use user interface to create analytical roadmaps based on the competitors, key stakeholders, and currently held market share to predict key drivers in the sales of pharmaceuticals [143], thus helping marketing executives allocate resources for the maximum market share gain, reversing poor sales and enabling them to anticipate where to make investments. 

### 3.4. Example Cases of AI in Drug Chemical Synthesis

The following example cases of AI in drug chemical synthesis highlight the use of artificial intelligence and ML in drug chemical synthesis. They explore the application of DL models in de novo drug design, the prediction of reaction outcomes, and the optimization of synthetic routes. The integration of AI with chemical knowledge and data-driven approaches has the potential to accelerate the drug discovery process and facilitate the design of new drug molecules. The paper by Paul D. et al. [14] is focused on the application of DL in drug discovery and medicinal chemistry, including the synthesis of new drug molecules. It explores the use of generative models and RL for de novo drug design and optimization.

Jiménez-Luna et al. [24] describe the use of AI in synthetic organic chemistry, specifically in reaction prediction, retrosynthesis, and reaction optimization. They discuss the integration of ML algorithms with chemical knowledge for efficient and automated synthesis planning. Their paper introduces DeepChem, a deep learning toolkit designed for drug discovery tasks. It covers various applications, including the generation of novel chemical structures, predicting chemical properties, and optimizing synthetic routes. The paper highlights the potential of deep learning in accelerating the drug discovery process. It also presents ML models for predicting the outcomes of organic reactions, including the yield and regioselectivity. It discusses the use of reaction fingerprints and molecular descriptors as input features for predicting reaction outcomes. The study demonstrates the potential of ML in guiding synthetic chemistry efforts.

### 3.5. Case Examples of AI Used in Drug Screening

These examples provide insights into the application of AI in drug screening and discovery. They highlight the use of AI algorithms for identifying novel compounds, designing new drug candidates, and predicting protein structures. The integration of AI in drug screening processes can help accelerate the discovery of potential drug candidates and optimize the drug discovery pipeline. 

Paul D et al. [14] discuss the integration of AI in various sectors of the pharmaceutical industry, including drug discovery and development. They highlight the use of AI in drug screening and repurposing, reducing the human workload, and accelerating the achievement of targets. Their paper also discusses the ongoing challenges and the potential ways to overcome them, along with the future of AI in the pharmaceutical industry.

Blanco-González et al. [3] present case studies demonstrating the successful application of AI in drug discovery. They showcase examples where AI algorithms have been used to identify novel compounds for cancer treatment, inhibitors for specific proteins involved in diseases like Alzheimer’s, and potential drugs for combating COVID-19. Their paper highlights the ability of AI to accelerate the drug discovery process and enable the development of more effective medications. It explores the impact of AI on the drug discovery process, with a focus on the design of novel compounds. It discusses how AI-based approaches, such as deep learning algorithms, can rapidly and efficiently design new drug candidates with desirable properties and activities. The paper also mentions the use of AI in structural biology, such as the development of AlphaFold, a software platform that uses AI to predict protein structures. This advancement has the potential to revolutionize personalized medicine and drug discovery.

Discovering drugs through AI models is a topic discussed on nexocode.com [144]. By analyzing large amounts of data, AI models can detect correlations that were previously unknown. This technology is being utilized in drug discovery to identify new drugs, design drug candidates, and assist in preclinical testing. AI is also being used to screen chemical repositories, identify new drug targets, and aid in computer-assisted molecular design.

A recent study showcased the use of AI in material discovery, as seen on unibas.ch [145]. The study’s author, a doctoral student, utilized quantum mechanics to generate predictions for thousands of crystals that had randomly determined chemical compositions. These predictions served as training data for statistical ML models. The ML models demonstrated an accuracy level equivalent to standard quantum mechanical approaches, but with the added benefit of speed. In a day, ML models can provide predictions, while quantum mechanical calculations would take a supercomputer over 20 million hours.

An interesting example is found on popularmechanics.com [146], where researchers used AI to develop a new type of compressible material. They did not have to rely on trial-and-error experimentation because they used existing research and AI algorithms to create new designs. This approach made the material discovery process much faster and allowed scientists to explore more design possibilities. The research also showed how important data-driven methods are in materials science. AI can suggest new approaches and designs as long as there are enough accurate data available.

The use of AI in the field of medicine goes beyond discovering new materials. According to ibm.com [147], AI has the potential to support precision medicine by providing patients with real-time recommendations that are tailored to their medical history, preferences, and needs. AI-powered virtual assistants are available 24/7 to provide healthcare information and address patient queries. Furthermore, AI is employed in medical imaging to detect diseases like breast cancer with comparable accuracy to human radiologists. Additionally, AI can help clinicians make sense of vast numbers of medical images and data, making them more manageable and enhancing patient care.

Healthcare is an ever-evolving field that is exploring the potential of AI in various medical domains. A comprehensive overview of AI in healthcare can be found on en.wikipedia.org [148]. In oncology, AI is used for cancer diagnosis, risk stratification, the molecular characterization of tumors, and drug discovery. For instance, AI algorithms have shown high accuracy in identifying breast cancer and prostate cancer. AI techniques such as artificial neural networks and Bayesian networks are used to diagnose and classify diseases. With the assistance of electronic health records, AI can analyze large amounts of data and help doctors with a patient’s diagnosis and treatment. Physics-inspired ML approaches are also being explored to enhance medical diagnostic approaches and perform biomarker analysis.

To benchmark and test AI applications in the health domain, the United Nations (WHO/ITU) has established the ITU-WHO Focus Group on Artificial Intelligence for Health (FG-AI4H). They are working on various cases for its use such as in assessing breast cancer risk, guiding antivenom selection, and diagnosing skin lesions.

The market potential of AI applications in drug discovery and development is immense. AI has emerged as a powerful tool in the pharmaceutical industry, revolutionizing the way new drugs are discovered, developed, and brought to market. With its ability to analyze vast amounts of data, AI offers the potential to accelerate the drug development process, reduce costs, and improve the success rate of bringing new medicines to patients.

Pharmaceutical companies, research institutions, and startups are increasingly investing in AI technologies to streamline drug discovery processes and gain a competitive edge in the market. Partnerships between AI companies and pharmaceutical giants are becoming more common, fostering the development of innovative drug discovery platforms.

### 3.6. AI in Drug Discovery and Repurposing

In this section we provide information on different drugs discovered by means of AI, highlighting AI’s significant role in drug discovery, accelerating the identification of potential drug candidates and optimizing drug development processes. In the following, we present some drugs that have been discovered or aided by AI.

Zong et al. [149] explore a computational drug repurposing application by using electronic health records (EHRs) to discover new applications for approved or investigational drugs. Their review identified four themes: publication venues, data types and sources, data processing and prediction methods, and targeted diseases, validation, and released tools. The utilization of EHRs in drug repurposing is hindered by validation, accessibility, and understanding issues.

Yadi et al. [150] introduce guidelines on how to use AI for accelerating drug repurposing or repositioning. They present how to use AI models in precision medicine, and as an example, how AI models can accelerate COVID-19 drug repurposing. 

In the paper by Wang et al. [151], a novel scoring algorithm, DrugRepo, uses chemical and genomic data to repurpose drugs for various diseases. In total, 516 approved drugs have been repurposed for 545 diseases using DrugRepo. Hundreds of novel predicted compounds have the potential for matching ongoing clinical trials. 

Different research groups used AI algorithms to discover that *naproxen*, a commonly used pain reliever, has potential antiviral properties. An excellent illustration of an in silico and target-based technique for finding novel antivirals is naproxen. Lejal et al. used the X-ray structure of the RNA-free NP of H1N1 as a prototype to develop a structural-based modeling technique to find potential medications that target the nucleoprotein (NP) of the influenza A virus [152]. The non-steroidal anti-inflammatory medication naproxen, a well-known inhibitor of inducible cyclooxygenase type 2 (COX-2), was discovered using an in silico screening that was concentrated on a selected particular location of the NP structure. AI models identified this drug as potential inhibitors of a viral enzyme, providing valuable insights for future antiviral drug development [152]. In another study, *naproxen* demonstrated antiviral properties against SARS-CoV-2 using an in silico molecular docking analysis (using Autodock Vina) [153]. 

By using molecular modeling and virtual screening studies, Mostafa et al. tested the antiviral activity of anti-microbial and anti-inflammatory FDA-approved drugs, commonly prescribed to relieve respiratory symptoms, against SARS-CoV-2 [154]. The docking study was performed using OpenEye scientific software version 2.2.5. A virtual screening study illustrated that Azithromycin, Niclosamide, and Nitazoxanide bind to the main protease of SARS-CoV-2. 

*Baricitinib* is an oral Janus kinase (JAK)1/JAK2 inhibitor approved for the treatment of rheumatoid arthritis. The Eli Lilly and Incyte Corporation used AI algorithms to analyze large-scale datasets to predict its usefulness on COVID-19 infection through planned anti-cytokine effects and as an inhibitor of host cell viral propagation [155]. 

*DSP-1181* was Developed by Sumitomo Dainippon Pharma and Exscientia. It is a drug designed using AI algorithms [156]. It became the first AI-designed drug to enter human clinical trials for the treatment of obsessive-compulsive disorder (OCD). DSP-1181 is a long-acting, potent serotonin 5-HT_1A_ receptor agonist [156]. AI played a pivotal role in the drug’s discovery by rapidly analyzing vast datasets, identifying potential drug candidates, and optimizing the molecular structures to enhance their therapeutic effects. The successful progression of DSP-1181 to clinical trials showcases the potential of AI in expediting drug development for challenging neurological disorders [156].

*DSP-2230* was also Developed by Sumitomo Dainippon Pharma and Exscientia, by means of AI-design, and is being investigated for the treatment of OCD. AI algorithms were utilized to analyze biological data and identify the novel compounds that can modulate the specific brain receptors implicated in the disorder [157].

*Berzosertib* (formerly M6620, VX-970), developed by Merck KGaA and C4X Discovery [158], is a promising drug investigated for its applications in targeting the DNA repair mechanisms in various cancers. AI played a significant role in identifying Berzosertib as a potential candidate for cancer treatment [159]. Through the AI-driven analysis of genetic and molecular data, researchers could pinpoint specific DNA repair pathways that the drug could effectively target, making it a promising addition to cancer therapy.

*Halicin* is a revolutionary antibiotic discovered by MIT researchers through the application of AI models [160]. It demonstrated remarkable effectiveness against a wide range of bacterial infections, including drug-resistant strains [161]. AI enabled the screening of a vast number of chemical compounds to identify Halicin as a potent antibiotic. The discovery of Halicin highlights the potential of AI in quickly identifying new classes of antibiotics to combat the growing problem of antibiotic resistance.

*Evinacumab* is a drug developed by Regeneron Pharmaceuticals using AI [162]. The drug was identified as a potential therapy for homozygous familial hypercholesterolemia (a rare genetic disorder characterized by extremely high cholesterol levels), refractory hypercholesterolemia (both familial and non-familial), and severe hypertriglyceridaemia [162]. AI algorithms were utilized to analyze vast datasets of genetic and biochemical information to identify the specific targets and pathways involved in cholesterol regulation. This helped researchers identify evinacumab as a monoclonal antibody that binds to and blocks an enzyme called ANGPTL3, thereby reducing cholesterol levels in affected individuals [162].

The Amarin Corporation developed *Icosapent Ethyl* to reduce cardiovascular risk [163]. The AI-driven analysis of clinical trial data helped demonstrate the drug’s effectiveness in reducing cardiovascular events in patients with high triglyceride levels, leading to its approval by regulatory authorities. Icosapent Ethyl is a high-purity prescription form of EPA ethyl ester approved by the US Food and Drug Administration at a dose of 4 g/day as an adjunct to diet to reduce plasma triglyceride levels in adults with severe hypertriglyceridemia [164]. 

LegoChem Biosciences, using AI models, developed *Delpazolid* (LCB-010371) as a potential antibiotic with effectiveness against drug-resistant bacteria, including Methicillin-resistant Staphylococcus aureus (MRSA) and Vancomycin-resistant Enterococci (VRE) [165]. AI-driven drug discovery methods were employed to identify Delpazolid as a novel antibiotic candidate. LegoChem Biosciences utilizes its ConjuAll and Legochemisty platform technologies to discover and develop drugs.

InSilico Medicine has used their AI platform Pharma.AI™ to develop *INS018-055* to help treat idiopathic pulmonary fibrosis [166]. Targeting the discoidin domain receptor 1 (DDR1) is how INS-08055 works. DDR1, an epithelial cell-expressed pro-inflammatory receptor tyrosine kinase involved in fibrosis, is activated by collagen and inhibited by INS-08055. The medication candidate, which is delivered orally and intravenously, improves illness symptoms by inhibiting DDR1 [166].

Developed by Merck & Co., *Pembrolizumab* (*Keytruda*) is an AI-discovered drug used for the treatment of various cancers, including melanoma, lung cancer, and head and neck cancers [167]. 

The UK Medicines and Healthcare Products Regulatory Agency (MHRA) has received a clinical trial application from BenevolentAI for *BEN-8744*, a small molecule phosphodiesterase 10 (PDE10) inhibitor that is intended to treat ulcerative colitis [168].

Sotorasib (AMG 510), developed by Amgen, is an AI-optimized drug used for the treatment of non-small cell lung cancer with specific genetic mutations. AI algorithms were used to analyze cancer genomic data and identify potential compounds that selectively inhibit the mutated protein responsible for tumor growth [169].

The above-mentioned drug examples highlight the diverse applications of AI in drug discovery and development, from optimizing drug structures and formulations to identifying novel targets and repurposing existing drugs for new indications. AI technologies continue to revolutionize the pharmaceutical industry, enabling more efficient and targeted drug development processes that hold the potential to transform patient care and treatment outcomes.

## 4. The Market Prospects of AI-Based Drug Development

Pharmaceutical companies are converting to AI solutions to reduce the financial costs and potential losses connected with virtual screening. Between 2015 and 2018, the AI market grew from USD 200 to USD 700 million, and by 2024, it is expected to reach USD 5 billion [170]. An expected 40% increase from 2017 to 2024 suggests that AI may reshape the pharmaceutical and medical industries. Many pharmaceutical companies have invested in AI or are continuing to do so, as well as collaborating with AI service providers to develop critical medical equipment. Examples of collaboration are given in Table 4.

## 5. Challenges and Limitations in AI-Assisted Drug Discovery

The use of AI to aid in the discovery of new materials holds immense potential in developing materials with specific properties for diverse applications. However, there are challenges and limitations that need to be acknowledged and addressed to ensure its effective implementation [3].

One of the main challenges highlighted in our paper is related to the quality and quantity of data. AI models require a large volume of high-quality data for training. In materials science, obtaining comprehensive and dependable datasets can be difficult due to the vast number of materials and properties that can be taken into consideration. Incomplete or inaccurate data can result in biased or erroneous predictions.

Therefore, reliable and high-quality data are the foundation of AI-assisted research. Industries must ensure that the data used for training AI models are precise, representative, and related to the specific research objectives. It is crucial to address data limitations, such as biases or incomplete datasets, to avoid skewed results and unreliable conclusions.

When it comes to discovering new materials, there are several important factors to consider. Firstly, data representation is key. This involves transforming complex material data, like crystal structures or chemical compositions, into formats that AI models can understand. It is important to develop representations that capture essential features while remaining interpretable [171].

Algorithm selection and development is also crucial. The appropriate AI algorithms for a material’s discovery will depend on the specific problem at hand. Developing custom algorithms that can handle the unique challenges of material data, such as high dimensionality and non-linear relationships, requires expertise in both AI and materials science.

While AI models can predict material properties with impressive accuracy, interpreting the underlying reasons for these predictions remains a challenge. Understanding the relationship between the input features and output properties is essential for guiding further research and design.

Finally, effective material discovery often requires the integration of domain-specific knowledge, such as quantum mechanics or thermodynamics. Combining AI techniques with specialized scientific insights is essential to ensure accurate predictions and meaningful discoveries [14].

When training data are biased, AI models may not perform well when presented with new materials or conditions. It is crucial to ensure diversity and representativeness in the training dataset to avoid this issue. However, researchers may face limitations in accessing a powerful computing infrastructure, which is often necessary for resource-intensive tasks, such as simulating material properties at different scales or conducting high-throughput calculations. Additionally, the vast and intricate search space of potential materials can pose a challenge when trying to find the optimal candidate. Advanced optimization techniques are required to explore this space efficiently [172].

It is also important to validate AI model predictions through experimental testing, which can be a time-consuming and costly process. Moreover, discovering new materials with advanced properties raises ethical and safety concerns. AI-assisted discoveries must be safe, environmentally friendly, and compliant with regulations. Industries must establish ethical guidelines that ensure responsible data usage, transparent algorithms, and the fair treatment of all stakeholders to address these concerns. Overall, AI-assisted research presents exciting opportunities but also requires the careful consideration of these issues [173]. When incorporating AI tools into the material discovery process, researchers must adjust their workflows and thinking. Cooperation between materials scientists and AI specialists is crucial for the optimal usage of these tools. AI-assisted material discovery offers exciting possibilities for revolutionizing the field by speeding up the identification of materials with the desired properties. However, the challenges and limitations mentioned above stress the importance of interdisciplinary collaboration, better data organization, innovative algorithms, and ethical and scientific considerations. Overcoming these obstacles will lead to the successful integration of AI in material discovery and ultimately fuel innovation across various industries [174]. 

Industries that handle sensitive or proprietary data should prioritize data security and implement strong measures to safeguard information against unauthorized access and breaches. It is crucial to balance the benefits of AI-powered insights with data security concerns. To ensure the reliability and applicability of AI-generated insights across various contexts, rigorous validation procedures are essential. Since AI-assisted research often requires significant computational resources, including powerful hardware and significant energy consumption, industries need to assess their computational needs and allocate resources effectively. Industries should implement strategies to identify and mitigate biases in AI models that may inadvertently perpetuate the biases present in the training data. In regulated industries, AI-assisted research must comply with relevant regulations and standards. To reap the full benefits of AI-driven research outcomes, industries must commit to long-term strategies that require continuous investment in updating models, refining algorithms, and adapting to evolving research needs. In fostering a collaborative environment, industries must encourage researchers to work alongside AI systems, leveraging their strengths to make more informed decisions and discoveries, and augmenting human capabilities [3]. To sum up, utilizing AI in research can lead to groundbreaking discoveries and drive innovation in various industries. However, it is crucial to carefully consider important factors such as data quality, ethics, expertise, security, and collaboration to ensure successful implementation. By thoughtfully addressing these considerations, industries can fully utilize the potential of AI to revolutionize their research practices and achieve unprecedented levels of discovery and innovation [3]. 

## 6. Future Directions and Opportunities in AI-Assisted Drugs Discovery

The global AI-in-drug discovery market is projected to witness significant growth in the coming years. Advancements in AI technologies, such as ML, natural language processing, and deep learning, have led to the development of sophisticated algorithms capable of analyzing complex biological data and predicting the interactions between drugs and their targets.

AI-driven platforms are being used for the virtual screening of large compound libraries, identifying potential drug candidates with higher accuracy and efficiency than traditional methods. These platforms can also optimize lead compounds and predict their pharmacokinetic and toxicity profiles, aiding in the selection of the most promising candidates for further development.

Furthermore, AI is transforming drug design by enabling the generation of novel molecular structures with specific properties. AI-generated molecules can be tailored to target specific diseases and biological pathways, offering new opportunities for precision medicine.

As regulatory agencies, such as the FDA, continue to embrace AI applications in drug development, the market potential of AI in the pharmaceutical industry is expected to grow further. However, challenges remain, including data privacy concerns, the validation of AI models, and the integration of AI with existing drug development processes.

Moreover, AI’s potential extends beyond drug discovery and development. It also plays a critical role in clinical trials, patient stratification, and the real-time monitoring of treatment responses, enabling personalized medical approaches that enhance patient outcomes.

The use of advanced simulation and modeling techniques in AI has the potential to revolutionize the drug discovery process, making it more efficient and effective. With AI, it is possible to model and simulate biological networks, which deepens our understanding of disease mechanisms and helps in the discovery of new drugs [175]. 

The use of AI in drug discovery has many benefits. Traditional approaches face obstacles such as high costs, inefficiency, and lengthy timelines. AI can address these issues by automating and optimizing processes, resulting in more efficient and effective drug discovery. This enables the discovery of novel drug candidates that may have been overlooked using traditional approaches. AI leverages advancements in biology and computing to develop cutting-edge algorithms for drug discovery, leveling the playing field and enabling more researchers to access powerful computational tools for drug development. Furthermore, AI models have a higher predictive power, reducing the potential for false positives in drug screening. By carefully designing the assay parameters, AI can identify meaningful interactions and prioritize promising targets for further investigation [176]. 

The use of AI in drug discovery shows potential by moving drug screening from the lab to a virtual environment, resulting in faster screening and the ability to identify promising targets without extensive effort and manpower. However, implementing advanced simulation and modeling techniques in AI for drug discovery poses challenges such as the data quality, interpretability of AI models, and the need for validation and reproducibility. Despite these challenges, AI has the potential to improve drug discovery efficiency, enable unbiased exploration, and enhance its predictive power, leading to reduced costs and an increased likelihood of successful drug candidates. To successfully integrate AI in drug discovery, addressing these challenges and ensuring the reliability and interpretability of AI models is crucial.

## 7. Conclusions

The future of AI in drug discovery involves a closer integration with automation, which would allow AI systems to make decisions on compound design and synthesis without human input. This shift from an augmented drug design paradigm to an autonomous one has the potential to accelerate the drug discovery process and yield better starting points for drug development. Ultimately, the goal is to develop fully autonomous laboratories that can iterate through the design–make–test–analyze cycle of drug discovery on their own. This could lead to faster and more efficient drug discovery processes, with AI systems suggesting and testing new compounds autonomously. However, there are challenges to overcome, such as proving the reliability and reproducibility of AI-assisted findings. Furthermore, the availability of robust datasets and investments in AI technology are important considerations for the future adoption and success of AI in drug discovery. 

The major challenge for the medical industry while developing a new drug is its increased costs and reduced efficiency. ML approaches and recent developments in DL come with great opportunities to reduce this cost, increase efficiency, and save time during the drug discovery and development process. Even though there are some obstacles and a tremendous amount of work to be conducted to incorporate AI tools in the drug discovery cycle, concretely, despite these challenges, AI has already demonstrated promising results in discovering new combinations of materials for drug discovery, accelerating the search for drug candidates, optimizing drug formulation and delivery, improving target identification, and enhancing virtual screening. The future outlook for AI in drug discovery is promising, with advancements expected in autonomous decision-making and the integration of AI with automation.

## Figures and Tables

**Table 1:** AI methods that are frequently utilized in drug discovery.

Class	Algorithms		Reference
**Supervised learning**	Regression analysis	MLR	[35]
		DT	[36]
		LR	[37]
	Classification	SVM	[38]
		CNN	[39]
		RNN	[40]
		GAN	[41]
**Unsupervised learning**	Clustering	k-means	[42]
		Hierarchical	[43]
	Dimensionality reduction	PCA	[44]
		t-SNE	[45]

**Table 2:** Collaborations between pharmaceutical companies and AI providers.

Pharmaceutical Company	AI Provider	Project Details
AstraZeneca	BenevolentAI	Selected the first AI-generated drug target for chronic kidney disease (CKD)
Pfizer	AI Technology	Used AI for COVID-19 vaccine trials and streamlined distribution. Also used AI and predictive analytics to modernize, streamline, and simplify the development of medicines.
Pfizer	XtalPi	Developed a hybrid physics and AI-powered software platform for the accurate molecular modeling of drug-like small molecules.
Pfizer	Insilico Medicine	Mined data for drug targets.
Pfizer	ConcertAI	Expanded partnership to improve study design and diversify clinical trials with the aid of AI.
Pfizer	Janssen Research and Development (Johnson & Johnson)	Collaborated to apply AI in the identification and selection of new targets and disease subsets to aid therapeutic programs.
Eli Lilly, Bayer, Bridge Biotherapeutics	Atomwise	Assisted in structure-based small-molecule drug discovery.
Boehringer Ingelheim	Google Quantum AI	Working together to leverage quantum computing to accelerate and optimize the discovery of future new medicines.
Boehringer Ingelheim	Insilico Medicine	Partnered to use AI technology in identifying potential therapeutic targets.
BMS	Exscientia	Contract led to the selection of an AI-designed immune-modulating drug candidate.
GSK	Exscientia	Developed the first-ever AI-powered treatment for COPD.
Roche, Sanofi, Bayer	Exscientia	Working with these big pharma players.
AstraZeneca	Eko, BERG, Renalytix AI, Mila-Quebec AI Institute	Using AI algorithms and supercomputers for drug discovery.
Optellum	J&J Lung Cancer Initiative	Applied AI-powered clinical decision support platform to transform early lung cancer treatment.

**Table 3:** AI tools/platforms already used in drug discovery.

Program/Platform	Description	Primary Use	Accession
**DeepChem**	Python-based AI system using MLP model	Candidate selection in drug discovery	https://github.com/deepchem/deepchem (accessed on 1 February 2024)
**DeepNeuralNetQSAR**	Python-based AI system	Can aid the detection of the molecular activity of compounds	https://github.com/Merck/DeepNeuralNet-QSAR (accessed on 1 February 2024)
**Chemputer**	Combination of Monte Carlo tree search and symbolic AI, including DNNs	Synthesize organic molecules	https://zenodo.org/record/1481731 (accessed on 1 February 2024)
**DeepTox**	AI system using DL	Chemical toxicity prediction	www.bioinf.jku.at/research/DeepTox (accessed on 1 February 2024)
**AlphaFold**	AI system using DL	Predicts the 3D structures of proteins	https://alphafold.ebi.ac.uk/ (accessed on 1 February 2024)
ORGANIC	Generative ML approaches and DNNs	Novel molecular materials	https://github.com/aspuru-guzik-group/ORGANIC (accessed on 1 February 2024)
PotentialNet	Neural networks, deep attention mechanisms and descriptor embeddings	The binding affinity of ligands in protein–ligand complexes.	https://www.genesistherapeutics.ai/platform.html (accessed on 1 February 2024)
Hit Dexter	ML technique, CNNs and ANNs	For predicting molecules that might respond to biochemical assays	http://hitdexter2.zbh.uni-hamburg.de (accessed on 1 February 2024)
DeltaVina	ML algorithms, including XGBoost and random forest	Scoring protein–ligand binding affinity	https://github.com/chengwang88/deltavina (accessed on 1 February 2024)
Neural graph fingerprint	CNNs	Predict properties of novel molecules	https://github.com/HIPS/neural-fingerprint (accessed on 1 February 2024)
GastroPlus	AI and predictive modeling	For pharmaceutical products (dosage form) in many animal models	https://www.simulations-plus.com/software/gastroplus/# (accessed on 1 February 2024)

**Table 4:** Collaborations of pharmaceutical companies with AI providers/developers for various applications.

Company	AI Use	Collaboration with the Pharmaceutical Industry	Application/Agents for Clinical Trials
IBM Watson Health Cambridge	AI for evaluating clinical and health-related data	Novartis	Real-time patient monitoring to improve breast cancer patient intervention outcomes
		Pfizer	Accelerating immuno-oncology medication discovery efforts
Benevolent AI	AI-enabled Judgement Augmented Cognition System (JACS) for developing new drugs effective against neurodegenerative diseases	Janssen	Such partnership will lead to the advancement of new medicinal molecules.
	Using AI, new clinical lead agents for chronic renal diseases are being developed.	AstraZeneca	During Phase 2b clinical trials, a drug candidate was assessed as a primary agent for treating chronic renal diseases.
Microsoft	AI for image processing and therapeutic interventions using cells and genes	Novartis	Creating an AI innovation lab to improve medication research and commercialization processes
Numerate	AI-enabled drug design for oncology and gastrointestinal specialties	Takeda	Phase 1 clinical trial of drug S48168 for Ryanodine Receptor 2
		Servier	Drug development for conditions of the central nervous system, the digestive system, and cancer
Owkin	Clinical testing by means of ML	Roche	Created and improved the Owkin’s Studio platform using artificial intelligence
XtalPi	A target identification and validation package integrating quantum mechanics and ML techniques	Pfizer	Preparation and improvement of crystalline drug candidate entities for use in early drug screening
Exscientia	AI-enabled drug discovery and lead refinement	Sanofi	Agent DSP-1181 is currently undergoing Phase I clinical testing. Advancement of the Centaur ChemistTM drug discovery AI system Drug discovery in obsessive-convulsive disorder
		Merck and BenevolentAI	New clinical development drug candidates in key therapeutic areas of oncology, neurology, and immunology
Atomwise	AI-enabled structural modeling	Lilly	Agent BBT-401 in Phase 2 of clinical testing
		Bridge Biotherapeutics	Augmentation of Pellino Inhibitor Pipeline Agent BBT-401 evaluated in Phase-2a of clinical testing
Sensyne Health	Clinical AI schemes	Bayer	Created and improved the specialized clinical AI technology suite for Sensyne Health.

## Data Availability

Not applicable.

## Abbreviations

AI	Artificial intelligence
DL	Deep learning
ML	Machine learning
ANN	Artificial neural network
CT	Computed Tomography Scan
MRI	Magnetic resonance imaging
ML	Machine Learning
KronRLS	Kronecker-regularized least squares
DTBA	Drug target binding affinity
GPCRs	G protein-coupled receptors
MLR	Multiple linear regression
DT	Drug target
LR	Logistic regression
SVM	Support vector machine
CNN	Convolution neural network
RNN	Recurrent neural network
GAN	Generative adversarial network
PCA	Principal-component analysis
t-SNE	t-distributed stochastic neighbor embedding
SVR	Support vector regression
Lasso	Least absolute shrinkage and selection operator
PBAEs	poly(beta-amino ester)s
DFT	High-throughput density functional theory
ADMET	Absorption, distribution, metabolism, excretion, and toxicity
QSAR	Quantitative structure-activity relationship
NLP	Natural language processing

## References

[1] Kaul V., Enslin S., Gross S.A. (2020). History of artificial intelligence in medicine. Gastrointest. Endosc..

[2] Rudrapal M., Kirboga K.K., Abdalla M., Maji S. (2024). Explainable artificial intelligence-assisted virtual screening and bioinformatics approaches for effective bioactivity prediction of phenolic cyclooxygenase-2 (COX-2) inhibitors using PubChem molecular fingerprints. Mol. Divers..

[3] Blanco-González A., Cabezón A., Seco-González A., Conde-Torres D., Antelo-Riveiro P., Piñeiro Á., Garcia-Fandino R. (2023). The Role of AI in Drug Discovery: Challenges, Opportunities, and Strategies. Pharmaceuticals.

[4] Malik P., Pathania M., Rathaur V.K. (2019). Overview of artificial intelligence in medicine. J. Fam. Med. Prim. Care.

[5] Briganti G., Le Moine O. (2020). Artificial Intelligence in Medicine: Today and Tomorrow. Front. Med..

[6] Yoon H.J., Jeong Y.J., Kang H., Jeong J.E., Kang D.-Y. (2019). Medical Image Analysis Using Artificial Intelligence. Prog. Med. Phys..

[7] Wu J., Chen J., Cai J. (2021). Application of Artificial Intelligence in Gastrointestinal Endoscopy. J. Clin. Gastroenterol..

[8] Kaur T., Diwakar A., Mirpuri P., Tripathi M., Chandra P.S., Gandhi T.K. (2021). Artificial Intelligence in Epilepsy. Neurol. India.

[9] Sohail A. (2023). Genetic Algorithms in the Fields of Artificial Intelligence and Data Sciences. Ann. Data Sci..

[10] Lee S., Kim H.-S. (2021). Prospect of Artificial Intelligence Based on Electronic Medical Record. J. Lipid Atheroscler..

[11] Contreras I., Vehi J. (2018). Artificial Intelligence for Diabetes Management and Decision Support: Literature Review. J. Med. Internet Res..

[12] Davis C.R., Murphy K.J., Curtis R.G., Maher C.A. (2020). A Process Evaluation Examining the Performance, Adherence, and Acceptability of a Physical Activity and Diet Artificial Intelligence Virtual Health Assistant. Int. J. Environ. Res. Public Health.

[13] Bhatt T.K., Nimesh S. (2021). The Design and Development of Novel Drugs and Vaccines: Principles and Protocols.

[14] Paul D., Sanap G., Shenoy S., Kalyane D., Kalia K., Tekade R.K. (2021). Artificial intelligence in drug discovery and development. Drug Discov. Today.

[15] Mathai N., Chen Y., Kirchmair J. (2020). Validation strategies for target prediction methods. Brief. Bioinform..

[16] Gupta R., Srivastava D., Sahu M., Tiwari S., Ambasta R.K., Kumar P. (2021). Artificial intelligence to deep learning: Machine intelligence approach for drug discovery. Mol. Divers..

[17] Yaseen B.T., Kurnaz S. (2023). Drug–target interaction prediction using artificial intelligence. Appl. Nanosci..

[18] Kumar R., Sharma A., Siddiqui M.H., Tiwari R.K. (2018). Prediction of Drug-Plasma Protein Binding Using Artificial Intelligence Based Algorithms. Comb. Chem. High Throughput Screen..

[19] Romeo-Guitart D., Forés J., Herrando-Grabulosa M., Valls R., Leiva-Rodríguez T., Galea E., González-Pérez F., Navarro X., Petegnief V., Bosch A. (2018). Neuroprotective Drug for Nerve Trauma Revealed Using Artificial Intelligence. Sci. Rep..

[20] Rifaioglu A.S., Atas H., Martin M.J., Cetin-Atalay R., Atalay V., Doğan T. (2019). Recent applications of deep learning and machine intelligence on in silico drug discovery: Methods, tools and databases. Brief. Bioinform..

[21] Zhang O., Zhang J., Jin J., Zhang X., Hu R., Shen C., Cao H., Du H., Kang Y., Deng Y. (2023). ResGen is a pocket-aware 3D molecular generation model based on parallel multiscale modelling. Nat. Mach. Intell..

[22] Gentile F., Yaacoub J.C., Gleave J., Fernandez M., Ton A.-T., Ban F., Stern A., Cherkasov A. (2022). Artificial intelligence–enabled virtual screening of ultra-large chemical libraries with deep docking. Nat. Protoc..

[23] Carpenter K.A., Huang X. (2018). Machine Learning-based Virtual Screening and Its Applications to Alzheimer’s Drug Discovery: A Review. Curr. Pharm. Des..

[24] Jiménez-Luna J., Grisoni F., Schneider G. (2020). Drug discovery with explainable artificial intelligence. Nat. Mach. Intell..

[25] Selvaraj C., Chandra I., Singh S.K. (2022). Artificial intelligence and machine learning approaches for drug design: Challenges and opportunities for the pharmaceutical industries. Mol. Divers..

[26] Sadybekov A.V., Katritch V. (2023). Computational approaches streamlining drug discovery. Nature.

[27] Boobier S., Hose D.R.J., Blacker A.J., Nguyen B.N. (2020). Machine learning with physicochemical relationships: Solubility prediction in organic solvents and water. Nat. Commun..

[28] Fagerholm U., Hellberg S., Spjuth O. (2021). Advances in Predictions of Oral Bioavailability of Candidate Drugs in Man with New Machine Learning Methodology. Molecules.

[29] Zhang L., Zhang H., Ai H., Hu H., Li S., Zhao J., Liu H. (2018). Applications of Machine Learning Methods in Drug Toxicity Prediction. Curr. Top. Med. Chem..

[30] Ashburn T.T., Thor K.B. (2004). Drug repositioning: Identifying and developing new uses for existing drugs. Nat. Rev. Drug Discov..

[31] Rani P., Dutta K., Kumar V. (2022). Artificial intelligence techniques for prediction of drug synergy in malignant diseases: Past, present, and future. Comput. Biol. Med..

[32] Gaweda A.E., Aronoff G.R., Brier M.E., Saggi S.J., Salifu M.O. (2022). Use of Artificial Intelligence/Machine Learning for Individualization of Drug Dosing in Dialysis Patients. Technological Advances in Care of Patients with Kidney Diseases.

[33] Vadapalli S., Abdelhalim H., Zeeshan S., Ahmed Z. (2022). Artificial intelligence and machine learning approaches using gene expression and variant data for personalized medicine. Brief. Bioinform..

[34] Vo T.H., Nguyen N.T.K., Kha Q.H., Le N.Q.K. (2022). On the road to explainable AI in drug-drug interactions prediction: A systematic review. Comput. Struct. Biotechnol. J..

[35] Dara S., Dhamercherla S., Jadav S.S., Babu C.M., Ahsan M.J. (2022). Machine Learning in Drug Discovery: A Review. Artif. Intell. Rev..

[36] Zeng X., Xiang H., Yu L., Wang J., Li K., Nussinov R., Cheng F. (2022). Accurate prediction of molecular properties and drug targets using a self-supervised image representation learning framework. Nat. Mach. Intell..

[37] Brito-Pacheco C., Brito-Loeza C., Martin-Gonzalez A. (2020). A regularized logistic regression based model for supervised learning. J. Algorithms Comput. Technol..

[38] Pisner D.A., Schnyer D.M., Mechelli A., Vieira S. (2020). Chapter 6—Support vector machine. Machine Learning.

[39] Smaldone A.M., Kyro G.W., Batista V.S. (2023). Quantum Convolutional Neural Networks for Multi-Channel Supervised Learning. arXiv.

[40] Kaur M., Mohta A. (2019). A Review of Deep Learning with Recurrent Neural Network. Proceedings of the 2019 International Conference on Smart Systems and Inventive Technology (ICSSIT).

[41] Lim H., Chon K.-W., Kim M.-S. (2023). Active learning using Generative Adversarial Networks for improving generalization and avoiding distractor points. Expert Syst. Appl..

[42] Sinaga K.P., Yang M.-S. (2020). Unsupervised K-Means Clustering Algorithm. IEEE Access.

[43] Lakshmi B.S.S.S., Ravi Kiran Varma P., Reddy V.S., Prasad V.K., Wang J., Reddy K.T.V. (2023). Machine Learning for Drug Discovery Using Agglomerative Hierarchical Clustering. Soft Computing and Signal Processing.

[44] Lever J., Krzywinski M., Altman N. (2017). Points of Significance: Principal component analysis. Nat. Methods.

[45] Balamurali M., Daya Sagar B.S., Cheng Q., McKinley J., Agterberg F. (2020). t-Distributed Stochastic Neighbor Embedding. Encyclopedia of Mathematical Geosciences.

[46] Rickert C.A., Lieleg O. (2022). Machine learning approaches for biomolecular, biophysical, and biomaterials research. Biophys. Rev..

[47] Stern M., Arinze C., Perez L., Palmer S.E., Murugan A. (2020). Supervised learning through physical changes in a mechanical system. Proc. Natl. Acad. Sci. USA.

[48] Usama M., Qadir J., Raza A., Arif H., Yau K.A., Elkhatib Y., Hussain A., Al-Fuqaha A. (2019). Unsupervised Machine Learning for Networking: Techniques, Applications and Research Challenges. IEEE Access.

[49] Talevi A., Morales J.F., Hather G., Podichetty J.T., Kim S., Bloomingdale P.C., Kim S., Burton J., Brown J.D., Winterstein A.G. (2020). Machine Learning in Drug Discovery and Development Part 1: A Primer. CPT Pharmacomet. Syst. Pharmacol..

[50] Zhou Z., Li X., Zare R.N. (2017). Optimizing Chemical Reactions with Deep Reinforcement Learning. ACS Cent. Sci..

[51] Kramer O., Kramer O. (2016). Scikit-Learn. Machine Learning for Evolution Strategies.

[52] Imambi S., Prakash K.B., Kanagachidambaresan G.R., Prakash K.B., Kanagachidambaresan G.R. (2021). PyTorch. Programming with TensorFlow: Solution for Edge Computing Applications.

[53] Gulli A., Pal S. (2017). Deep Learning with Keras.

[54] Priya S., Tripathi G., Singh D.B., Jain P., Kumar A. (2022). Machine learning approaches and their applications in drug discovery and design. Chem. Biol. Drug Des..

[55] Pandis N. (2016). Linear regression. Am. J. Orthod. Dentofac. Orthop..

[56] Taskinen J., Yliruusi J. (2003). Prediction of physicochemical properties based on neural network modelling. Adv. Drug Deliv. Rev..

[57] Suprapto S., Nikmah Y.L. (2023). Ridge and Lasso Regression for Feature Selection of Overlapping Ibuprofen and Paracetamol UV Spectra. Moroc. J. Chem..

[58] Roozbeh M., Arashi M., Hamzah N.A. (2020). Generalized Cross-Validation for Simultaneous Optimization of Tuning Parameters in Ridge Regression. Iran. J. Sci. Technol. Trans. Sci..

[59] Kim Y., Hao J., Mallavarapu T., Park J., Kang M. (2019). Hi-LASSO: High-Dimensional LASSO. IEEE Access.

[60] Abdulhafedh A. (2022). Comparison between Common Statistical Modeling Techniques Used in Research, Including: Discriminant Analysis vs Logistic Regression, Ridge Regression vs LASSO, and Decision Tree vs Random Forest. Open Access Libr. J..

[61] Patel L., Shukla T., Huang X., Ussery D.W., Wang S. (2020). Machine Learning Methods in Drug Discovery. Molecules.

[62] Peña-Guerrero J., Nguewa P.A., García-Sosa A.T. (2021). Machine learning, artificial intelligence, and data science breaking into drug design and neglected diseases. WIREs Comput. Mol. Sci..

[63] Ozdemir S., Susarla D. (2018). Feature Engineering Made Easy: Identify Unique Features from Your Dataset in Order to Build Powerful Machine Learning Systems.

[64] Chauhan N.K., Singh K. (2018). A Review on Conventional Machine Learning vs Deep Learning. Proceedings of the 2018 International Conference on Computing, Power and Communication Technologies (GUCON).

[65] Janiesch C., Zschech P., Heinrich K. (2021). Machine learning and deep learning. Electron. Mark..

[66] Gong D., Ben-Akiva E., Singh A., Yamagata H., Est-Witte S., Shade J.K., Trayanova N.A., Green J.J. (2022). Machine learning guided structure function predictions enable in silico nanoparticle screening for polymeric gene delivery. Acta Biomater..

[67] Taye M.M. (2023). Understanding of Machine Learning with Deep Learning: Architectures, Workflow, Applications and Future Directions. Computers.

[68] Choudhary K., Kalish I., Beams R., Tavazza F. (2017). High-throughput Identification and Characterization of Two-dimensional Materials using Density functional theory. Sci. Rep..

[69] Friedman R. (2022). Computational studies of protein–drug binding affinity changes upon mutations in the drug target. WIREs Comput. Mol. Sci..

[70] Varadharajan V., Arumugam G.S., Shanmugam S. (2022). Isatin-based virtual high throughput screening, molecular docking, DFT, QM/MM, MD and MM-PBSA study of novel inhibitors of SARS-CoV-2 main protease. J. Biomol. Struct. Dyn..

[71] Joseph Sahayarayan J., Soundar Rajan K., Nachiappan M., Prabhu D., Guru Raj Rao R., Jeyakanthan J., Hossam Mahmoud A., Mohammed O.B., Morgan A.M.A. (2020). Identification of potential drug target in malarial disease using molecular docking analysis. Saudi J. Biol. Sci..

[72] Faris A., Ibrahim I.M., Hadni H., Elhallaoui M. (2023). High-throughput virtual screening of phenylpyrimidine derivatives as selective JAK3 antagonists using computational methods. J. Biomol. Struct. Dyn..

[73] Sohlenius-Sternbeck A.-K., Terelius Y. (2022). Evaluation of ADMET Predictor in Early Discovery Drug Metabolism and Pharmacokinetics Project Work. Drug Metab. Dispos..

[74] Öeren M., Walton P.J., Hunt P.A., Ponting D.J., Segall M.D. (2021). Predicting reactivity to drug metabolism: Beyond P450s—Modelling FMOs and UGTs. J. Comput. Aided Mol. Des..

[75] Rydberg P., Jørgensen F.S., Olsen L. (2014). Use of density functional theory in drug metabolism studies. Expert Opin. Drug Metab. Toxicol..

[76] Smirnova A., Yablonskiy M., Petrov V., Mitrofanov A. (2023). DFT Prediction of Radiolytic Stability of Conformationally Flexible Ligands. Energies.

[77] Alzain A.A., Elbadwi F.A., Alsamani F.O. (2022). Discovery of novel TMPRSS2 inhibitors for COVID-19 using in silico fragment-based drug design, molecular docking, molecular dynamics, and quantum mechanics studies. Inform. Med. Unlocked.

[78] Bhatnagar R., Sardar S., Beheshti M., Podichetty J.T. (2022). How can natural language processing help model informed drug development?: A review. JAMIA Open.

[79] Zheng S., Dharssi S., Wu M., Li J., Lu Z., Larson R.S., Oprea T.I. (2019). Text Mining for Drug Discovery. Bioinformatics and Drug Discovery.

[80] Dumitriu A., Molony C., Daluwatte C., Sikos L.F., Seneviratne O.W., McGuinness D.L. (2021). Graph-Based Natural Language Processing for the Pharmaceutical Industry. Provenance in Data Science: From Data Models to Context-Aware Knowledge Graphs.

[81] Study of the Drug-Related Adverse Events with the Help of Electronic Health Records and Natural Language Processing—ProQuest. https://www.proquest.com/openview/e0e053ffe5b850bd656912f47db18b77/1?pq-origsite=gscholar&cbl=5444811.

[82] Corcoran C.M., Mittal V.A., Bearden C.E., Gur R.E., Hitczenko K., Bilgrami Z., Savic A., Cecchi G.A., Wolff P. (2020). Language as a biomarker for psychosis: A natural language processing approach. Schizophr. Res..

[83] Jang G., Lee T., Hwang S., Park C., Ahn J., Seo S., Hwang Y., Yoon Y. (2018). PISTON: Predicting drug indications and side effects using topic modeling and natural language processing. J. Biomed. Inform..

[84] Piñero J., Bravo À., Queralt-Rosinach N., Gutiérrez-Sacristán A., Deu-Pons J., Centeno E., García-García J., Sanz F., Furlong L.I. (2017). DisGeNET: A comprehensive platform integrating information on human disease-associated genes and variants. Nucleic Acids Res..

[85] Szklarczyk D., Gable A.L., Lyon D., Junge A., Wyder S., Huerta-Cepas J., Simonovic M., Doncheva N.T., Morris J.H., Bork P. (2019). STRING v11: Protein–protein association networks with increased coverage, supporting functional discovery in genome-wide experimental datasets. Nucleic Acids Res..

[86] Szklarczyk D., Santos A., von Mering C., Jensen L.J., Bork P., Kuhn M. (2016). STITCH 5: Augmenting protein–chemical interaction networks with tissue and affinity data. Nucleic Acids Res..

[87] Salehi P., Chalechale A., Taghizadeh M. (2020). Generative Adversarial Networks (GANs): An Overview of Theoretical Model, Evaluation Metrics, and Recent Developments. arXiv.

[88] Blanchard A.E., Stanley C., Bhowmik D. (2021). Using GANs with adaptive training data to search for new molecules. J. Cheminformatics.

[89] Abbasi M., Santos B.P., Pereira T.C., Sofia R., Monteiro N.R.C., Simões C.J.V., Brito R.M.M., Ribeiro B., Oliveira J.L., Arrais J.P. (2022). Designing optimized drug candidates with Generative Adversarial Network. J. Cheminformatics.

[90] Zhang Z., Li F., Guan J., Kong Z., Shi L., Zhou S., Razavi-Far R., Ruiz-Garcia A., Palade V., Schmidhuber J. (2022). GANs for Molecule Generation in Drug Design and Discovery. Generative Adversarial Learning: Architectures and Applications.

[91] Barcelos M.P., Gomes S.Q., Federico L.B., Francischini I.A.G., Hage-Melim L.I.d.S., Silva G.M., de Paula da Silva C.H.T., Taft C.A., de Lazaro S.R. (2022). Lead Optimization in Drug Discovery. Research Topics in Bioactivity, Environment and Energy: Experimental and Theoretical Tools.

[92] Lin E., Lin C.-H., Lane H.-Y. (2020). Relevant Applications of Generative Adversarial Networks in Drug Design and Discovery: Molecular De Novo Design, Dimensionality Reduction, and De Novo Peptide and Protein Design. Molecules.

[93] Wu B., Li L., Cui Y., Zheng K. (2022). Cross-Adversarial Learning for Molecular Generation in Drug Design. Front. Pharmacol..

[94] Zhao L., Wang J., Pang L., Liu Y., Zhang J. (2020). GANsDTA: Predicting Drug-Target Binding Affinity Using GANs. Front. Genet..

[95] Gan J., Liu J., Liu Y., Chen S., Dai W., Xiao Z.-X., Cao Y. (2023). DrugRep: An automatic virtual screening server for drug repurposing. Acta Pharmacol. Sin..

[96] Tripathi S., Augustin A.I., Dunlop A., Sukumaran R., Dheer S., Zavalny A., Haslam O., Austin T., Donchez J., Tripathi P.K. (2022). Recent advances and application of generative adversarial networks in drug discovery, development, and targeting. Artif. Intell. Life Sci..

[97] Sureyya Rifaioglu A., Nalbat E., Atalay V., Jesus Martin M., Cetin-Atalay R., Doğan T. (2020). DEEPScreen: High performance drug–target interaction prediction with convolutional neural networks using 2-D structural compound representations. Chem. Sci..

[98] Cai C., Wang S., Xu Y., Zhang W., Tang K., Ouyang Q., Lai L., Pei J. (2020). Transfer Learning for Drug Discovery. J. Med. Chem..

[99] Zhuang D., Ibrahim A.K. (2021). Deep Learning for Drug Discovery: A Study of Identifying High Efficacy Drug Compounds Using a Cascade Transfer Learning Approach. Appl. Sci..

[100] Miller E., Hansch C. (1967). Structure-Activity Analysis of Tetrahydrofolate Analogs Using Substituent Constants and Regression Analysis. J. Pharm. Sci..

[101] Schneider N., Jäckels C., Andres C., Hutter M.C. (2008). Gradual in Silico Filtering for Druglike Substances. J. Chem. Inf. Model..

[102] Jorissen R.N., Gilson M.K. (2005). Virtual Screening of Molecular Databases Using a Support Vector Machine. J. Chem. Inf. Model..

[103] Hou T., Wang J., Li Y. (2007). ADME Evaluation in Drug Discovery. 8. The Prediction of Human Intestinal Absorption by a Support Vector Machine. J. Chem. Inf. Model..

[104] Wu Z., Ramsundar B., Feinberg E.N., Gomes J., Geniesse C., Pappu A.S., Leswing K., Pande V. (2018). MoleculeNet: A benchmark for molecular machine learning. Chem. Sci..

[105] Ma J., Sheridan R.P., Liaw A., Dahl G.E., Svetnik V. (2015). Deep Neural Nets as a Method for Quantitative Structure–Activity Relationships. J. Chem. Inf. Model..

[106] Yang K., Swanson K., Jin W., Coley C., Eiden P., Gao H., Guzman-Perez A., Hopper T., Kelley B., Mathea M. (2019). Analyzing Learned Molecular Representations for Property Prediction. J. Chem. Inf. Model..

[107] Ragoza M., Hochuli J., Idrobo E., Sunseri J., Koes D.R. (2017). Protein–Ligand Scoring with Convolutional Neural Networks. J. Chem. Inf. Model..

[108] Wang C., Zhang Y. (2017). Improving scoring-docking-screening powers of protein–ligand scoring functions using random forest. J. Comput. Chem..

[109] Segler M.H.S., Preuss M., Waller M.P. (2018). Learning to Plan Chemical Syntheses. Nature.

[110] Coley C.W., Thomas D.A., Lummiss J.A.M., Jaworski J.N., Breen C.P., Schultz V., Hart T., Fishman J.S., Rogers L., Gao H. (2019). A robotic platform for flow synthesis of organic compounds informed by AI planning. Science.

[111] Xu Y., Lin K., Wang S., Wang L., Cai C., Song C., Lai L., Pei J. (2019). Deep learning for molecular generation. Future Med. Chem..

[112] Elton D.C., Boukouvalas Z., Fuge M.D., Chung P.W. (2019). Deep learning for molecular design—A review of the state of the art. Mol. Syst. Des. Eng..

[113] Dana D., Gadhiya S.V., St. Surin L.G., Li D., Naaz F., Ali Q., Paka L., Yamin M.A., Narayan M., Goldberg I.D. (2018). Deep Learning in Drug Discovery and Medicine; Scratching the Surface. Molecules.

[114] Kitchin R., McArdle G. (2016). What makes Big Data, Big Data? Exploring the ontological characteristics of 26 datasets. Big Data Soc..

[115] Reker D., Schneider G. (2015). Active-learning strategies in computer-assisted drug discovery. Drug Discov. Today.

[116] DeepChem. https://github.com/deepchem/deepchem.

[117] Merck/DeepNeuralNet-QSAR. Merck Sharp & Dohme Corp. a Subsidiary of Merck & Co., Inc. https://github.com/Merck/DeepNeuralNet-QSAR.

[118] Keenan G. (2018). Croningp/ChemputerSoftware: Chemputer First Release.

[119] Mayr A., Klambauer G., Unterthiner T., Hochreiter S. (2016). DeepTox: Toxicity Prediction using Deep Learning. Front. Environ. Sci..

[120] AlphaFold. https://www.deepmind.com/research/highlighted-research/alphafold.

[121] Jumper J., Evans R., Pritzel A., Green T., Figurnov M., Ronneberger O., Tunyasuvunakool K., Bates R., Žídek A., Potapenko A. (2021). Highly accurate protein structure prediction with AlphaFold. Nature.

[122] ORGANIC The Matter Lab, Aspuru-Guzik Group Repo, 80 St. George Street Toronto, ON, M5S 3H6. https://github.com/aspuru-guzik-group/ORGANIC.

[123] Kwak H.S., An Y., Giesen D.J., Hughes T.F., Brown C.T., Leswing K., Abroshan H., Halls M.D. (2022). Design of Organic Electronic Materials with a Goal-Directed Generative Model Powered by Deep Neural Networks and High-Throughput Molecular Simulations. Front. Chem..

[124] Mostaghimi M., Rêgo C.R.C., Haldar R., Wöll C., Wenzel W., Kozlowska M. (2022). Automated Virtual Design of Organic Semiconductors Based on Metal-Organic Frameworks. Front. Mater..

[125] Chen P., Wang Y., Yan H., Gao S., Xu Z., Li Y., Mo Q., Huang J., Tao J., Pan G. (2020). 3DStructGen: An interactive web-based 3D structure generation for non-periodic molecule and crystal. J. Cheminform..

[126] Feinberg E.N., Sur D., Wu Z., Husic B.E., Mai H., Li Y., Sun S., Yang J., Ramsundar B., Pande V.S. (2018). PotentialNet for Molecular Property Prediction. ACS Cent. Sci..

[127] Seo S., Choi J., Park S., Ahn J. (2021). Binding affinity prediction for protein–ligand complex using deep attention mechanism based on intermolecular interactions. BMC Bioinform..

[128] Kandel J., Tayara H., Chong K.T. (2021). PUResNet: Prediction of protein-ligand binding sites using deep residual neural network. J. Cheminform..

[129] Wang C. DeltaVina. https://github.com/chengwang88/deltavina.

[130] Kumar S., Kim M. (2021). SMPLIP-Score: Predicting ligand binding affinity from simple and interpretable on-the-fly interaction fingerprint pattern descriptors. J. Cheminform..

[131] Yang C., Zhang Y. (2022). Delta Machine Learning to Improve Scoring-Ranking-Screening Performances of Protein–Ligand Scoring Functions. J. Chem. Inf. Model..

[132] Neural Graph Fingerprints Formerly: Harvard Intelligent Probabilistic Systems Group—Now at Princeton. https://github.com/HIPS/neural-fingerprint.

[133] Wen N., Liu G., Zhang J., Zhang R., Fu Y., Han X. (2022). A fingerprints based molecular property prediction method using the BERT model. J. Cheminform..

[134] GastroPlus^®^ PBPK & PBBM Modeling and Simulation. Simulations Plus. https://www.simulations-plus.com/software/gastroplus/.

[135] Hussain A., Afzal O., Yasmin S., Haider N., Altamimi A.S.A., Martinez F., Acree W.E., Ramzan M. (2023). Preferential Solvation Study of Rosuvastatin in the {PEG400 (1) + Water (2)} Cosolvent Mixture and GastroPlus Software-Based In Vivo Predictions. ACS Omega.

[136] Schneider P., Walters W.P., Plowright A.T., Sieroka N., Listgarten J., Goodnow R.A., Fisher J., Jansen J.M., Duca J.S., Rush T.S. (2020). Rethinking drug design in the artificial intelligence era. Nat. Rev. Drug Discov..

[137] Ertl P., Schuffenhauer A. (2009). Estimation of synthetic accessibility score of drug-like molecules based on molecular complexity and fragment contributions. J. Cheminform..

[138] Chaudhari R., Fong L.W., Tan Z., Huang B., Zhang S. (2020). An up-to-date overview of computational polypharmacology in modern drug discovery. Expert Opin. Drug Discov..

[139] Reddy A.S., Zhang S. (2013). Polypharmacology: Drug discovery for the future. Expert Rev. Clin. Pharmacol..

[140] Sirois C., Khoury R., Durand A., Deziel P.-L., Bukhtiyarova O., Chiu Y., Talbot D., Bureau A., Després P., Gagné C. (2021). Exploring polypharmacy with artificial intelligence: Data analysis protocol. BMC Med. Inform. Decis. Mak..

[141] Duch W., Swaminathan K., Meller J. (2007). Artificial Intelligence Approaches for Rational Drug Design and Discovery. Curr. Pharm. Des..

[142] Blasiak A., Khong J., Kee T. (2020). CURATE.AI: Optimizing Personalized Medicine with Artificial Intelligence. SLAS Technol. Transl. Life Sci. Innov..

[143] Baronzio G., Parmar G., Baronzio M. (2015). Overview of Methods for Overcoming Hindrance to Drug Delivery to Tumors, with Special Attention to Tumor Interstitial Fluid. Front. Oncol..

[144] Nexocode. https://nexocode.com/.

[145] Universität Basel. https://www.unibas.ch/de.html.

[146] Popular Mechanics—Product Reviews, How-To, Space, Military, Math, Science, and New Technology. Popular Mechanics. https://www.popularmechanics.com/.

[147] IBM—United Kingdom. https://www.ibm.com/uk-en.

[148] Main Page. Wikipedia, the Free Encyclopedia. https://en.wikipedia.org/w/index.php?title=Main_Page&oldid=1114291180.

[149] Zong N., Wen A., Moon S., Fu S., Wang L., Zhao Y., Yu Y., Huang M., Wang Y., Zheng G. (2022). Computational drug repurposing based on electronic health records: A scoping review. Npj Digit. Med..

[150] Zhou Y., Wang F., Tang J., Nussinov R., Cheng F. (2020). Artificial intelligence in COVID-19 drug repurposing. Lancet Digit. Health.

[151] Wang Y., Aldahdooh J., Hu Y., Yang H., Vähä-Koskela M., Tang J., Tanoli Z. (2022). DrugRepo: A novel approach to repurposing drugs based on chemical and genomic features. Sci. Rep..

[152] Lejal N., Tarus B., Bouguyon E., Chenavas S., Bertho N., Delmas B., Ruigrok R.W.H., Di Primo C., Slama-Schwok A. (2013). Structure-Based Discovery of the Novel Antiviral Properties of Naproxen against the Nucleoprotein of Influenza A Virus. Antimicrob. Agents Chemother..

[153] Terrier O., Dilly S., Pizzorno A., Chalupska D., Humpolickova J., Bouřa E., Berenbaum F., Quideau S., Lina B., Fève B. (2021). Antiviral Properties of the NSAID Drug Naproxen Targeting the Nucleoprotein of SARS-CoV-2 Coronavirus. Molecules.

[154] Mostafa A., Kandeil A., Elshaier Y.A.M.M., Kutkat O., Moatasim Y., Rashad A.A., Shehata M., Gomaa M.R., Mahrous N., Mahmoud S.H. (2020). FDA-Approved Drugs with Potent In Vitro Antiviral Activity against Severe Acute Respiratory Syndrome Coronavirus 2. Pharmaceuticals.

[155] Stebbing J., Krishnan V., de Bono S., Ottaviani S., Casalini G., Richardson P.J., Monteil V., Lauschke V.M., Mirazimi A., Youhanna S. (2020). Mechanism of baricitinib supports artificial intelligence-predicted testing in COVID-19 patients. EMBO Mol. Med..

[156] Farghali H., Kutinová Canová N., Arora M. (2021). The Potential Applications of Artificial Intelligence in Drug Discovery and Development. Physiol. Res..

[157] Evaluation of Safety, Tolerability & PK of DSP-2230 in Healthy Subjects. Health Research Authority. https://www.hra.nhs.uk/planning-and-improving-research/application-summaries/research-summaries/evaluation-of-safetytolerability-pk-of-dsp-2230-in-healthy-subjects/.

[158] Terranova N., Jansen M., Falk M., Hendriks B.S. (2021). Population pharmacokinetics of ATR inhibitor berzosertib in phase I studies for different cancer types. Cancer Chemother. Pharmacol..

[159] Plummer R., Dean E., Arkenau H.-T., Redfern C., Spira A.I., Melear J.M., Chung K.Y., Ferrer-Playan J., Goddemeier T., Locatelli G. (2022). A phase 1b study evaluating the safety and preliminary efficacy of berzosertib in combination with gemcitabine in patients with advanced non-small cell lung cancer. Lung Cancer.

[160] Almallah Z., El-Lababidi R., Shamout F., Doyle D.J. (2021). Artificial Intelligence: The New Alexander Fleming. Healthc. Inform. Res..

[161] Valavanidis A. Artificial Intelligence Application with Machine-Learning Algorithm Identified a Powerful Broad-Spectrum Antibiotic. http://chem-tox-ecotox.org/wp-content/uploads/2020/03/ANTIBIOTICS-HALICIN-ARTIFICIAL-INTELLIGENCE-2020.pdf.

[162] Markham A. (2021). Evinacumab: First Approval. Drugs.

[163] Miller M., Tokgozoglu L., Parhofer K.G., Handelsman Y., Leiter L.A., Landmesser U., Brinton E.A., Catapano A.L. (2022). Icosapent ethyl for reduction of persistent cardiovascular risk: A critical review of major medical society guidelines and statements. Expert Rev. Cardiovasc. Ther..

[164] Ballantyne C.M., Manku M.S., Bays H.E., Philip S., Granowitz C., Doyle R.T., Juliano R.A. (2019). Icosapent Ethyl Effects on Fatty Acid Profiles in Statin-Treated Patients with High Triglycerides: The Randomized, Placebo-controlled ANCHOR Study. Cardiol. Ther..

[165] kgi-admin. Delpazolid by LegoChem Biosciences for Tuberculosis: Likelihood of Approval. Pharmaceutical Technology. https://www.pharmaceutical-technology.com/data-insights/delpazolid-legochem-biosciences-tuberculosis-likelihood-of-approval/.

[166] Healthcare G. First Drug Created by AI Enters Clinical Trials. Clinical Trials Arena. https://www.clinicaltrialsarena.com/comment/first-drug-created-ai-enters-trials/.

[167] Evaxion Cleared by FDA to Begin Phase IIb Trial of Cancer Vaccine, Keytruda in Melanoma. Precision Medicine Online. https://www.precisionmedicineonline.com/cancer/evaxion-cleared-fda-begin-phase-iib-trial-cancer-vaccine-keytruda-melanoma.

[168] Arnold C. (2023). Inside the nascent industry of AI-designed drugs. Nat. Med..

[169] The Discovery of Amgen’s Novel Investigational KRAS(G12C) Inhibitor AMG 510 Published in Nature. https://www.amgen.com/newsroom/press-releases/2019/10/the-discovery-of-amgens-novel-investigational-krasg12c-inhibitor-amg-510-published-in-nature.

[170] Ghislaine PELLAT. Constantin Anghelache. Governance in the EU Member States in the Era of Big Data. In Proceedings of the 25th PGV Network Conference—International Scientific Conference, Bucharest, Romania, 12–13 September 2019. https://www.researchgate.net/profile/Grzegorz-Maciejewski/publication/335929459_Use_of_Big_Data_On_The_Food_Market_-_Areas_Applications_Examples/links/5d84bb29a6fdcc8fd6fda856/Use-of-Big-Data-On-The-Food-Market-Areas-Applications-Examples.pdf.

[171] Yang X., Wang Y., Byrne R., Schneider G., Yang S. (2019). Concepts of Artificial Intelligence for Computer-Assisted Drug Discovery. Chem. Rev..

[172] How to Navigate the Patenting Challenges of AI-Assisted Drug Discovery. https://www.pharmaceuticalonline.com/doc/how-to-navigate-the-patenting-challenges-of-ai-assisted-drug-discovery-0001.

[173] Freedman D.H. (2019). Hunting for New Drugs with AI. Nature.

[174] How AI Is Aiming at the Bad Math of Drug Development. Bloomberg.com, 29 November 2021. https://www.bloomberg.com/news/articles/2021-11-29/how-ai-is-aiming-at-the-bad-math-of-drug-development-quicktake.

[175] DiNuzzo M. (2022). How artificial intelligence enables modeling and simulation of biological networks to accelerate drug discovery. Front. Drug Discov..

[176] Fleming N. (2018). How artificial intelligence is changing drug discovery. Nature.

